# *Salmonella* effector SopB reorganizes cytoskeletal vimentin to maintain replication vacuoles for efficient infection

**DOI:** 10.1038/s41467-023-36123-w

**Published:** 2023-01-30

**Authors:** Shuangshuang Zhao, Qiuping Xu, Yanqin Cui, Su Yao, Sihui Jin, Qian Zhang, Zeyu Wen, Haihua Ruan, Xin Liang, Yanjie Chao, Sitang Gong, Philippe Sansonetti, Ke Wei, Hong Tang, Yaming Jiu

**Affiliations:** 1 Guangzhou Institute of Pediatrics, Guangzhou Women and Children’s Medical Center, Guangzhou Medical University, Guangzhou, 510623 China; 2 Unit of Cell Biology and Imaging Study of Pathogen Host Interaction, The Center for Microbes, Development and Health, CAS Key Laboratory of Molecular Virology and Immunology, Institut Pasteur of Shanghai, Chinese Academy of Sciences, Shanghai, 200031 China; 3 CAS Key Laboratory of Molecular Virology and Immunology, Institut Pasteur of Shanghai, Chinese Academy of Sciences, Shanghai, 200031 China; 4 Institute for Regenerative Medicine, Shanghai East Hospital, Shanghai Institute of Stem Cell Research and Clinical Translation, Shanghai Key Laboratory of Signaling and Disease Research, Frontier Science Center for Stem Cell Research, School of Life Sciences and Technology, Tongji University, Shanghai, 200092 China; 5 University of Chinese Academy of Sciences, Yuquan Road No. 19(A), Shijingshan District, Beijing, 100049 China; 6 Tianjin Key Laboratory of Food Science and Biotechnology, College of Biotechnology and Food Science, Tianjin University of Commerce, Tianjin, 300134 China; 7 Tsinghua-Peking Joint Center for Life Sciences, School of Life Sciences, Tsinghua University, Beijing, 100084 China

**Keywords:** Intermediate filaments, Cellular microbiology, Bacterial infection

## Abstract

A variety of intracellular bacteria modulate the host cytoskeleton to establish subcellular niches for replication. However, the role of intermediate filaments, which are crucial for mechanical strength and resilience of the cell, and in bacterial vacuole preservation remains unclear. Here, we show that *Salmonella* effector SopB reorganizes the vimentin network to form cage-like structures that surround *Salmonella*-containing vacuoles (SCVs). Genetic removal of vimentin markedly disrupts SCV organization, significantly reduces bacterial replication and cell death. Mechanistically, SopB uses its N-terminal Cdc42-binding domain to interact with and activate Cdc42 GTPase, which in turn recruits vimentin around SCVs. A high-content imaging-based screening identified that MEK1/2 inhibition led to vimentin dispersion. Our work therefore elucidates the signaling axis SopB-Cdc42-MEK1/2 as mobilizing host vimentin to maintain concrete SCVs and identifies a mechanism contributing to *Salmonella* replication. Importantly, Trametinib, a clinically-approved MEK1/2 inhibitor identified in the screen, displayed significant anti-infection efficacy against *Salmonella* both in vitro and in vivo, and may provide a therapeutic option for treating drug-tolerant salmonellosis.

## Abstract (web summary)

Vimentin is an intermediate filament protein responsible for maintaining cell shape. Here, the authors characterise the role of the *Salmonella* effector SopB on the vimentin network and its contribution to infection.

## Introduction

*Salmonella* is a major food-borne pathogen that causes millions of gastrointestinal and systemic diseases globally each year. Upon invasion into host cells, the majority of *Salmonella* replicates in a membrane-bound compartment known as the *Salmonella*-containing vacuole (SCV)^1,2^. SCV maturation relies on *Salmonella* pathogenicity island-1 (SPI-1)- and −2 (SPI-2)-encoded type 3 secretion systems (T3SS). SPI-1 encoded T3SS is mainly known as mediating bacterial invasion^3^, while SPI-2 encoded T3SS is commonly considered to be essential for bacterial replication and survival^4^. There is growing evidence that the function of effectors encoded by SPI-1 and SPI-2 is not limited to bacterial invasion and replication, respectively. For instance, SopB, a SPI-1 encoded effector, promotes bacterial entry via controlling actin cytoskeleton reorganization^5^. However, SopB has also been reported to be recruited to SCV to regulate its subcellular positioning through activation of the Rho/Rho kinase (ROCK)/myosin II pathway^5,6^. However, the underlying mechanisms of how *Salmonella* effectors and host interaction partners in SCV associated bacterial replication remains incompletely understood.

Emerging evidences suggest that cytoskeletal actin filaments reassembling and microtubule motor-based movement are crucial host cytoskeletal machinery for the oriented migration and positioning of SCV to the perinuclear region, for efficient replication^7–10^. Cytoskeleton is targeted by many bacterial virulence proteins, to promote bacteria internalization, trafficking, and provide structural support for SCV. For instance, SteC, a *Salmonella* kinase, is required for SPI-2-dependent F-actin remodelling^11^. In addition to actin and microtubule cytoskeleton, drastic remodeling of vimentin intermediate filaments (IFs) have been witnessed upon *Salmonella* infection, for maintaining SCV in the juxtanuclear area^12^. After positioning, *Salmonella* within SCVs starts to replicate, and the concrete SCV structure becomes critical for successful replication. However, it remains unclear whether concentrated SCV as an entirety contribute to bacterial replication, and how hyperplastic IFs regulate SCV and subsequent bacterial replication.

Vimentin is an abundant IFs protein that plays a key role in cell morphology, migration, and signaling^13–17^. On account of its non-essentiality, vimentin knockout mice are viable with mild defects in fertilization^18^, but display severe symptoms upon stimuli, such as chronic wounding and mechanical stretching^19^, suggesting that vimentin may play important roles in pathological processes. Interestingly, vimentin has been implicated during pathogen infection^20,21^. It can bind to the cell surface to facilitate certain bacterial invasion^22^, and is involved in triggering innate immune signaling upon bacterial lipopolysaccharide (LPS) stimuli^23^. Interaction between vimentin and effector protein IpaC is required for stable docking of *S*. flexneri to cells^24^. However, it remains unclear whether and how vimentin filaments, normally accumulating in the perinuclear region, regulate perinuclear anchored SCV for efficient *Salmonella* replication.

In this work, we demonstrated that *Salmonella* infection induced massive rearrangements of vimentin to encage SCV, and vimentin ablation led to dispersal of SCVs and decreased intracellular bacteria replication. Immunoprecipitation and mass spectrometry analysis identified the interaction between vimentin and bacterial effector SopB. Imaging-based screen and permutational and combinational assays revealed a SopB-Cdc42-MEK1/2 signaling axis that controls vimentin remodeling upon *Salmonella* infection, for SCV organization. In both cellular and mouse model, we identified that the MEK1/2 inhibitor Trametinib exhibited a significant anti-infection effect against *Salmonella*. Together, our findings underpin a key host-bacterial interplay mechanism and potential therapeutics for salmonellosis.

## Results

### Vimentin promotes *Salmonella* replication by maintaining concrete SCVs

**Fig. 1:**
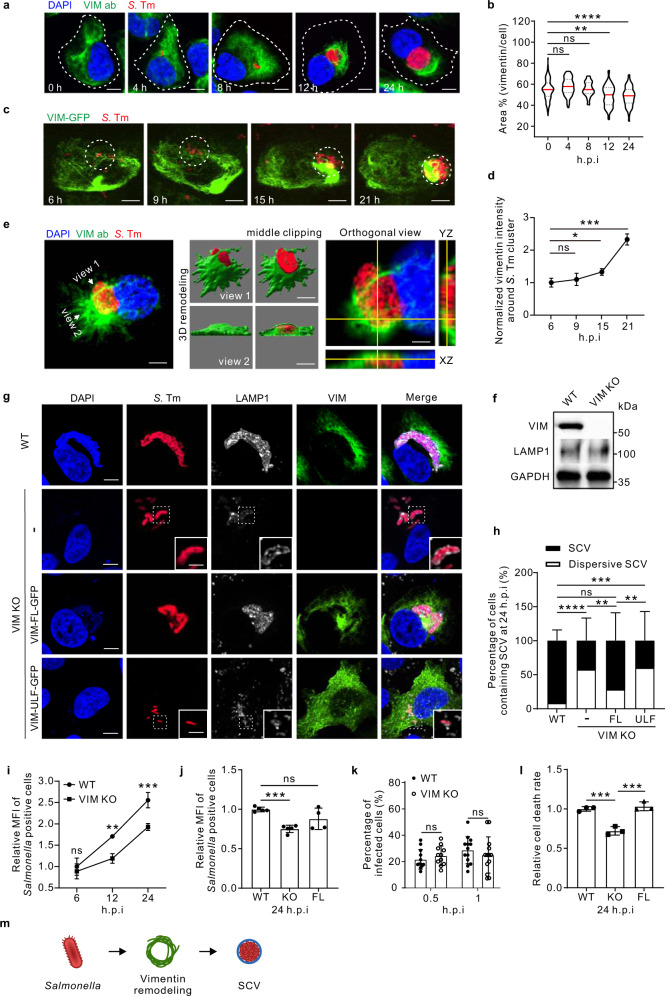
Host vimentin IFs promote the cytosolic replication of *Salmonella* by maintaining SCV morphology. **a** Immunofluorescence images of cells infected with *Salmonella* (MOI = 10). **b** Quantification of relative vimentin area versus cell area in (**a**) was measured. n = 30 views (60×/1.5 oil objective) from three independent experiments. **c** Time-lapse imaging of vimentin-GFP expressing cells infected with *Salmonella* (MOI = 10). **d** Quantification of normalized vimentin-GFP intensity around *Salmonella* cluster in (**c**) from three independent experiments. **e** Representative 3D-SIM super-resolution imaging and the cross-section showing the cage-like structure of vimentin around *Salmonella* (MOI = 10) at 24 *hpi*. **f** Western blotting of wild-type (WT) and vimentin knockout (VIM KO) cell lines. GAPDH serves as the loading control. **g** Immunofluorescence images of cells infected with *Salmonella* (MOI = 10) at 24 *hpi*. **h** Quantification of the percentage of SCV versus dispersive SCV in (**g**) was measured. *n* = 20 views (60×/1.5 oil objective) from three independent experiments. **i** Quantification of relative MFI values by FACS in WT and VIM KO cells infected with mCherry-tagged *Salmonella* (MOI = 50) from three independent experiments. **j** Quantification of relative MFI values by FACS in cells infected with mCherry-tagged *Salmonella* (MOI = 50) over three independent experiments. **k** Percentage of infected cells in WT and VIM KO background (MOI = 10). n = 20 views (60×/1.5 oil objective) from three independent experiments. **l** Quantification of the normalized cell death rate in cells infected with *Salmonella* (MOI = 10) at 24 *hpi* from three independent experiments. **m** Schematic diagram of *Salmonella* induced vimentin remodeling to surround SCV. White dash lines in (**a**) indicate the outline of the cells. Assays were conducted three times independently with similar results (**e** and **f**). Scale bars, 10 μm (**a**, **c**, the cell images in **e** and **g**) and 2 μm (the magnified images in **e** and **g**). Data are represented as mean ± SD. Statistics (ns, *p* > 0.05; *p < 0.05; **p < 0.01; ***p < 0.001; ****p < 0.0001): unpaired two-tailed Student’s *t*-test (**d**, **k**), one-way ANOVA with Dunnett’s analysis (**b**, **j** and **l**) or two-way ANOVA with Sidak’s analysis (**i**, **h**). Source data are provided as a Source Data file.

To study whether host cytoskeletal proteins respond to *Salmonella* infection, we examined the vimentin network using an established model with human osteosarcoma cells (U2OS) that express abundant cytoskeletal filaments and are highly permissive to bacterial infection^25,26^. A plasmid-borne *mCherry* gene was introduced into wild-type *Salmonella* (strain LT2) by electroporation for fluorescence visualization, which does not affect the pathogenicity, as confirmed by measuring the rate of cell death (Fig. S1a). In resting cells, endogenous vimentin filaments were perinuclearly localized and the typical filamentous structure radiated towards cell periphery (Fig. 1a). However, vimentin markedly rearranged from 12 h post infection (*hpi*) upon *Salmonella* infection (Fig. 1b). Vimentin formed compact aggregates, together with mCherry-tagged *Salmonella* within 24 *hpi*, without any obvious changes in cell size and morphology (Fig. 1a, b). Consistently, live-cell imaging showed that GFP-tagged vimentin gradually gathered from ~15 *hpi* and progressively intensified around bacterial clusters in infected cells (Fig. 1c,d; Movie S1). Bacteria multiplied in the perinuclear region by forming the *Salmonella*-containing vacuoles (SCVs)^12^. Taking advantage of the three-dimensional structured illumination microscopy (3D-SIM) powered super-resolution visualization, we observed that vimentin filaments formed a hollow cage-like structure wrapping SCVs (Fig. 1e). Similar SCV wrapping structure of vimentin filaments could also be observed using another *Salmonella* strain SL1344, excluding the possibility that this was a strain-specific phenomenon (Fig. S1b-e). In addition, heat-killed and paraformaldehyde (PFA)-fixed dead *Salmonella* failed to induce vimentin rearrangement (Fig. S1f,g), indicating that our observation is a bacteria-induced rather than a host-induced phenotype.

To determine whether there is a causal relationship between vimentin rearrangement and *Salmonella* infection, we used the CRISPR/Cas9 method to establish vimentin-knockout (KO) cells and the lentiviral system to construct vimentin KO-full length (FL) rescue cells (Fig. 1f; Fig. S1h). Intriguingly, bacteria displayed a piecemeal distribution in most vimentin KO cells, which was rarely seen in wild-type cells (Fig. 1g, h). Using late lysosome-associated LAMP1 protein as SCV marker^12^, we observed that the bacteria enclosing with LAMP1-positive vacuoles are scattered in vimentin KO cells (Fig. 1g). We designated these small vacuoles containing *Salmonella* as dispersive SCVs henceforth. Depletion of vimentin dramatically increased the proportion of dispersive SCVs from 8% up to 58%, which was rescued by overexpression of FL vimentin (Fig. 1g, h). Nevertheless, vimentin mutant only forming unit length filaments (ULF)^27^ or squiggles failed to restore normal SCV (Fig. 1g, h).

SCV is critical for *Salmonella* replication^11,12^. To address whether dispersive SCVs correlates with bacterial replication, we measured the intracellular bacterial loads by determining the mean fluorescence intensity (MFI) of *Salmonella* positive cells by flow cytometry FACS^28^. Significantly reduced MFI was observed in vimentin deficient cells from 12 *hpi* towards the later period of replication (Fig. 1i; Fig. S1j). Consistently, decreased MFI was also witnessed in vimentin deficiency fibroblast MEFs cells and macrophage RAW 264.7 cells (Fig. S1i,j), suggesting the compromised bacterial replication is not a cell-type specific phenotype, but rather a general regulation. Overexpression of exogenous FL vimentin partially restored bacterial replication at 24 *hpi* (Fig. 1j; Fig. S1j). Given that vimentin shrinking was evident after 12 *hpi* (Fig. 1a), we speculated that vimentin does not participate in the early phase of infection. Indeed, the proportions of infected cells were not different between wild-type and vimentin KO cells at 0.5 and 1 *hpi* (Fig. 1k). Moreover, *Salmonella* infection led to cell death in the late stage^29^, which was mitigated in vimentin-depleted, but not FL vimentin re-expressing cells (Fig. 1l). Taken together, these results showed that vimentin plays a critical role in *Salmonella* infection by maintaining normal SCVs morphology (Fig. 1m).

### Vimentin interacts with SopB of *Salmonella* to maintain concrete SCV during infection

**Fig. 2:**
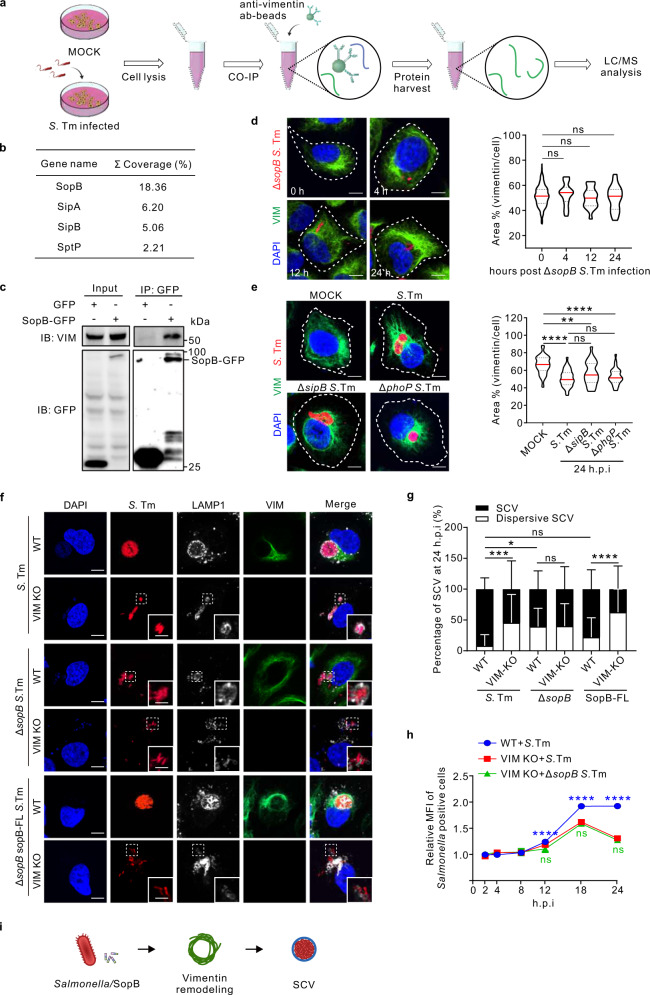
Vimentin interacts with SopB to facilitate the intracellular replication of *Salmonella* during infection. **a** Schematic diagram to detect vimentin interactome by precipitation and mass spectrometry. **b** List of the top *Salmonella* T3SS effectors interacting with vimentin from mass spectrometry. **c** Co-immunoprecipitation (Co-IP) assay followed by Western blotting to test for association of SopB-GFP with vimentin. SopB-GFP was enriched using anti-GFP agarose beads, and presence of vimentin or SopB was tested by Western blotting. Co-IP assays were conducted three times independently with similar results. **d** Immunofluorescence images of cells infected with Δ*sopB Salmonella* (MOI = 10). Quantification of the relative vimentin area versus cell area is shown in the right panel. **e** Immunofluorescence images of cells infected with *Salmonella* (WT, Δ*sipB*, Δ*phoP*) (MOI = 10) at 24 *hpi*. Quantification of the relative vimentin area versus cell area is shown in the right panel. **f** Immunofluorescence images of vimentin, LAMP1, and bacteria in WT and VIM KO cells infected with *Salmonella*, Δ*sopB Salmonella*, and full length sopB complemented Δ*sopB Salmonella* (Δ*sopB* sopB-FL *Salmonella*) (MOI = 10) at 24 *hpi*, respectively. **g** Quantification of the percentage of SCV versus dispersive SCV in (**f**) was measured. **h** Quantification of relative MFI values by FACS in WT and VIM KO cells infected with mCherry-tagged *Salmonella* or Δs*opB Salmonella* (MOI = 50) from three independent experiments. **i** Schematic diagram of SopB from *Salmonella* induced vimentin remodeling to surround SCV. White dash lines in (**d** and **e**) indicate the outline of the cells. *n* = 20 views (60×/1.5 oil objective) from three independent experiments in (**d**, **e** and **g**). Scale bar, 10 μm (**d**, **e** and the cell images in **f**) and 5 μm (the magnified images in **f**). Data are represented as mean ± SD. Statistics (ns, *p* > 0.05; *p < 0.05; **p < 0.01; ***p < 0.001; ****p < 0.0001): one-way ANOVA with Dunnett’s analysis (right panel of **d** and **e**) or two-way ANOVA with Sidak’s analysis (**g** and **h**). Source data are provided as a Source Data file.

To explore how vimentin functions in *Salmonella* infection, we performed co-immunoprecipitation (co-IP) with vimentin antibody in *Salmonella* infected cells to identify *Salmonella* proteins interacting with vimentin (Fig. 2a). Mass spectrometry analysis of vimentin interacting components revealed that hundreds host proteins started or lost interaction with vimentin post *Salmonella* infection (Fig. S2a–c), and importantly, several *Salmonella* type III secretion system (T3SS) effectors associated with vimentin (Fig. 2b). A top candidate SopB was further studied because of its localization to SCV and involvement in the replication of *Salmonella*^30–32^. Co-IP of exogenously expressed SopB-GFP with vimentin confirmed the interaction (Fig. 2c). Moreover, we generated a *Salmonella* strain with native chromosomally FLAG-tagged SopB (Fig. S2d). Upon infection, FLAG-tagged SopB was secreted into host cells and displayed intimate co-localization with endogenous vimentin (Fig. S2e).

To address whether SopB-vimentin interaction is required for SCV maintenance, we utilized a SopB-deleted *Salmonella* strain (Δ*sopB S*. Tm, Fig. S2f) and tagged it with mCherry, which shows no pathogenic difference, compared to non-tagged Δ*sopB* bacteria (Fig. S2g). Δ*sopB* strain showed similar percentage of infected cells at 0.5 and 1 *hpi* in wild-type and vimentin depletion cells (Fig. S2h). However, vimentin failed to aggregate post Δ*sopB* bacterial infection in several cell lines, including U2OS (Fig. 2d), MEFs (Fig. S2i, j) and RAW 264.7 (Fig. S2k, l). To eliminate the possibility of any other effector in this process, a control set of SipB (another SPI-1 effector) and PhoP (a SPI-2 master regulator whose deficiency disables SPI-2 effectors) KO bacteria were used. Vimentin still aggregated around SCV post Δ*sipB* or Δ*phoP* strain infection (Fig. 2e), indicating that they are not able to trigger vimentin-dependent replication. We thus concluded that SopB is primarily required for vimentin aggregation. In addition, increased proportion of dispersive SCVs was observed in cells infected with Δ*sopB* strain compared to the wild-type parental strain, but not with SopB-FL re-expressing strain (Fig. 2f, g), while the absence of vimentin did not augment dispersive SCVs infected with Δ*sopB* bacteria compared to wild-type *Salmonella* (Fig. 2f, g). Consistently, absence of SopB no longer further affected bacterial replication in vimentin KO cells (Fig. 2h), indicating that SopB is the causal factor directing vimentin aggregation for bacterial replication in SCVs. Together, we identified that bacterial SopB plays a critical role in vimentin-mediated maintaining of concrete SCV during *Salmonella* replication (Fig. 2i).

### N-terminal Cdc42-binding domain of SopB interacts with and activates host Cdc42 to control vimentin rearrangement and subsequent SCV maintenance

**Fig. 3:**
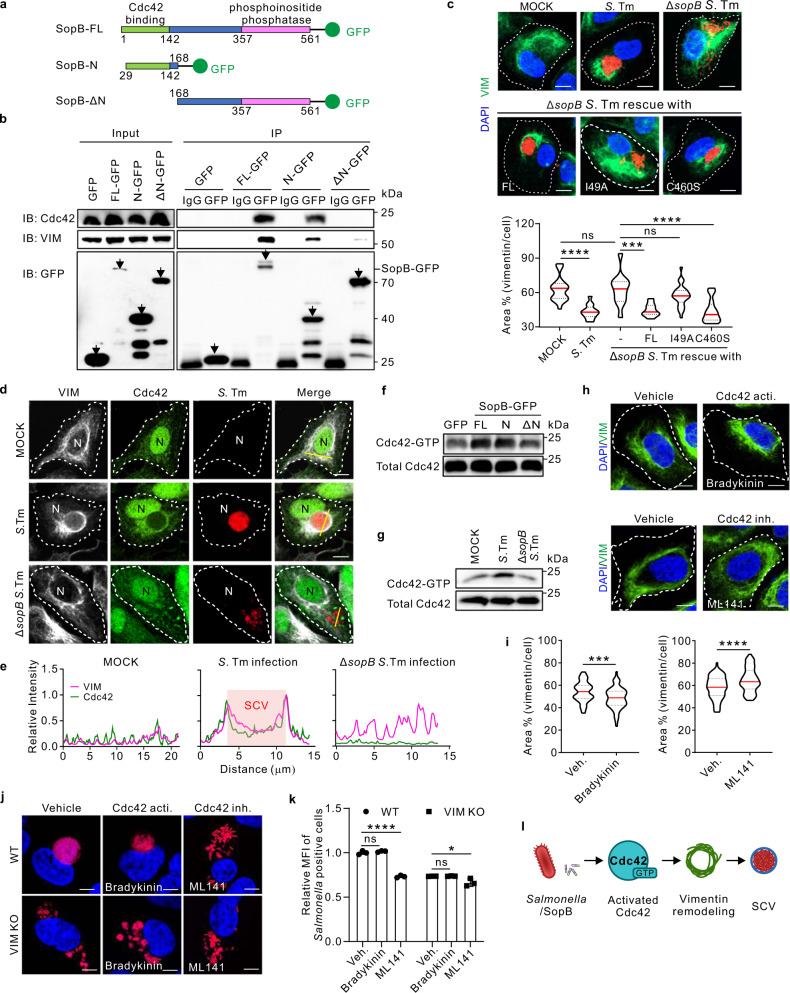
The binding and activation of Cdc42 by the N-terminus of bacterial SopB are essential for vimentin-dependent intracellular replication of *Salmonella*. **a** Domain structure and constructed truncations of SopB. GFP was fused to the C-terminus. **b** Co-IP assay followed by Western blotting to test for association of different truncations of SopB-GFP with Cdc42 and vimentin. **c** Immunofluorescence images of cells infected with *Salmonella*, Δ*sopB Salmonella*, sopB-FL or sopB^I49A^ (I49A) or sopB^C460S^ (C460S) complemented Δ*sopB Salmonella* (MOI = 10) at 24 *hpi*. Quantification of the relative vimentin area is shown in the bottom panel. **d** Immunofluorescence images of cells infected with *Salmonella* and Δ*sopB Salmonella* (MOI = 10) at 24 *hpi*. N indicated the position of the nucleus. **e** Relative fluorescence intensity profile of vimentin and Cdc42 signaling based on the yellow line in (**d**). Pink square indicated the position of SCV. **f** Western blotting analysis to detect the active level of Cdc42 upon sopB constructs transfection. **g** Western blotting analysis to detect the active level of Cdc42 upon *Salmonella* or Δ*sopB Salmonella* infection (MOI = 10) at 24 *hpi*. **h** Immunofluorescence images of cells treated with the activator bradykinin and inhibitor ML141 of Cdc42, respectively. **i** Quantification of the relative vimentin area in (**h**) was measured. **j** Immunofluorescence images of cells infected with *Salmonella* (MOI = 10) at 24 *hpi*, treated with bradykinin and ML141, respectively. **k** Quantification of relative MFI values by FACS in cells infected with mCherry-tagged *Salmonella* (MOI = 50) at 24 *hpi*, treated with bradykinin and ML141 from three independent experiments. **l** Schematic diagram of activated Cdc42 by *Salmonella*/SopB induced vimentin remodeling to surround SCV. White dash lines in (**c**, **d** and **h**) indicate the outline of the cells. *n* = 20 views (60×/1.5 oil objective) from three independent experiments in (**c** and **i**). Assays were conducted three times independently with similar results (**b**, **d**–**g**). Scale bars, 10 μm (**c**, **d**, **h** and **j**). Data are represented as mean ± SD. Statistics (ns, *p* > 0.05; ***p < 0.001; ****p < 0.0001): one-way ANOVA with Dunnett’s analysis (**c** and **i**) or two-way ANOVA with Sidak’s analysis (**k**). Source data are provided as a Source Data file.

SopB possesses two functional domains, a Cdc42-binding domain in the N-terminus (29-142), and a phosphoinositide phosphatase domain in the C-terminus (357-561)^33^. Co-IP analyses of vimentin and GFP-tagged SopB in full-length (SopB-FL), N-terminus (SopB-N) and N-terminus truncated (SopB-ΔN) constructs (Fig. 3a) showed that only SopB-ΔN lost the ability to interact with Cdc42 and vimentin (Fig. 3b). Therefore, the N-terminus of SopB is critical for its interaction with both Cdc42 and vimentin. To confirm the functional domain of SopB required for the rearrangement of vimentin, SopB^I49A^ and SopB^C460S^ mutants were introduced to Δ*sopB S*. Tm, which disrupts interaction with Cdc42^33^ and abrogates phosphatidylinositol phosphatase activity of SopB^34^, respectively. In accordance with SopB-FL re-expressing Δ*sopB* strain, SopB^C460S^ induced vimentin reorganization, whereas SopB^I49A^ failed to (Fig. 3c), indicating that the interaction between SopB-N terminal and Cdc42 is essential to trigger vimentin rearrangement, while the phosphoinositide phosphatase activity of SopB is dispensable.

To examine whether Cdc42 is involved in SCV maintenance regulated by SopB and vimentin, we examined the subcellular localization and the active level of Cdc42 upon bacterial infection. In agreement with vimentin reorganization, Cdc42 became enriched around SCVs post wild-type *S*. Tm infection, whereas it showed diffused distribution in un-infected or Δ*sopB S*. Tm infected condition (Fig. 3d, e). Monitoring the GTP-binding form of Cdc42 showed that overexpression of SopB-FL and SopB-N, but not SopB-ΔN, significantly activated Cdc42 (Fig. 3f; Fig. S3a). This was further substantiated by Δ*sopB* infection, where Cdc42 activity was reduced as compared to wild-type bacteria at 24 *hpi* (Fig. 3g; Fig. S3b). These results therefore suggested that the N-terminus of SopB not only binds but also activates Cdc42. Cdc42 activation can collapse the vimentin network in fibroblasts^35,36^. Consistently, we found that shrinking or spreading of vimentin was modulated by Cdc42 activator Bradykinin or inhibitor ML141, respectively (Fig. S3c; Fig. 3h, i), without inducing cellular toxicity (Fig. S3d, e). Moreover, the effects of these chemicals on vimentin distribution were verified by overexpressing the dominant negative (DN) Cdc42 mutant (Fig. S3g).

Moreover, treatment with Cdc42 inhibitor ML141 (Fig. 3j; Fig. S3h) or overexpression of Cdc42-DN (Fig. S3i) disassembled SCVs giving rise to increased proportion of dispersive SCV in wild-type, but not in vimentin KO cells (Fig. 3j; Fig. S3h, i). Similarly, Cdc42 inhibitor ML141 remarkably reduced MFI measured by FACS in wild type cells, but only slightly decreased in vimentin KO cells (Fig. 3k), indicating that the effect of Cdc42 activity on SCV maintenance is primarily dependent on vimentin. Together with the finding that SopB activates Cdc42, these results suggest that SopB-Cdc42-vimentin signaling axis is critical for maintaining concrete SCV during *Salmonella* infection (Fig. 3l).

### MEK1/2, enhanced by SopB, is required for vimentin rearrangement and SCV maintenance

**Fig. 4:**
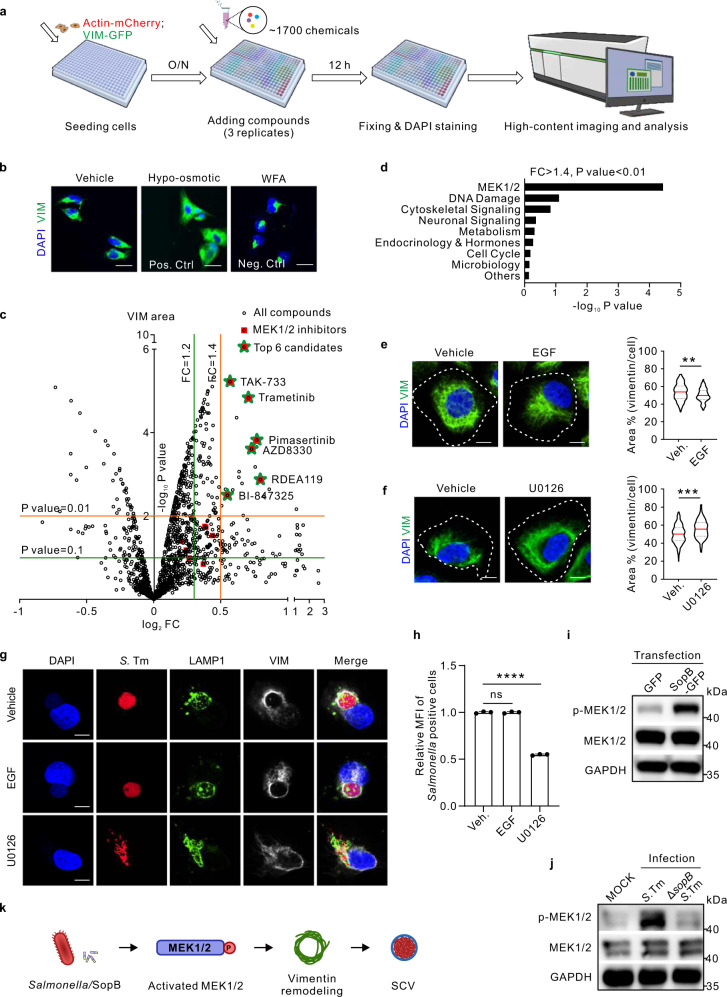
An imaging-based screen identifies MEK1/2 pathway to control vimentin rearrangement and subsequent intracellular replication of *Salmonella*. **a** Schematic diagram of the imaging-based high-content drug screen. **b** Immunofluorescence images of vimentin in cells treated with hypo-osmotic shock (as a positive control) and WFA (as a negative control), respectively. **c** Volcano plot revealed candidates inducing vimentin rearrangement from the screen. Red squares represent candidates belonging to MEK1/2 inhibitors. Green stars represent the top six candidates belonging to MEK1/2 inhibitors to disperse vimentin (FC > 1.4, *P*-value < 0.01). **d** Enrichment of signaling pathways of the top candidates (FC > 1.4, *P*-value < 0.01) from the screen. **e**,**f**, Immunofluorescence images of cells treated with the commercial activator EGF (**e**) and inhibitor U0126 (**f**) of MEK1/2 for 24 h, respectively. Quantification of the relative vimentin area versus cell area is shown in the right panel. *n* = 20 views (60×/1.5 oil objective) from three independent experiments. **g** Immunofluorescence images of cells infected with *Salmonella* (MOI = 10) at 24 *hpi*, treated with EGF and U0126 for 24 h, respectively. **h** Quantification of relative MFI values by FACS in cells infected with mCherry-tagged *Salmonella* (MOI = 50) at 24 *hpi*, treated with EGF and U0126, respectively, from three independent experiments. **i** Western blotting analysis to detect the phosphorylation level of MEK1/2 upon sopB-GFP transfection. **j** Western blotting analysis to detect the phosphorylation level of MEK1/2 upon *Salmonella* or Δ*sopB Salmonella* infection (MOI = 10) at 24 *hpi*. GAPDH serves as the loading control (**i**, **j**). **k** Schematic diagram of activated MEK1/2 by *Salmonella*/SopB induced vimentin remodeling to surround SCV. White dash lines in (**e** and **f**) indicate the outline of the cells. Assays were conducted three times independently with similar results (**b**, **i** and **j**). Scale bars, 10 μm (**e**, **f** and **g**) and 20 μm (**b**). Data are represent**e**d as mean ± SD. Statistics (ns, *p* > 0.05; **p < 0.01; ***p < 0.001; ****p < 0.0001): unpaired two-tailed Student’s *t*-test (**c**, right panel of **e** and **f**), EASE score (**d**) or one-way ANOVA with Dunnett’s analysis (**h**). Source data are provided as a Source Data file.

Aforementioned vimentin-dependent maintaining of SCVs prompted us to search for vimentin-targeting compounds that might suppress *Salmonella* infection. We therefore set up an imaging-based scoring system (ratios of vimentin area versus cell area) to screen a small molecule library of ~1700 clinically-approved and/or tested drug compounds with known targets (Fig. 4a) in cells that stably expressed vimentin-GFP and Actin-mCherry (Fig. S4a). Hypo-osmotic shock that disperses vimentin^37^ and Withaferin A (WFA) causing vimentin aggregation^38^ were used as positive and negative controls, respectively (Fig. 4b). Nuclei were counted to exclude cytotoxic effect (Fig. S4b). Calculated volcano plot of the screen results revealed 19 compounds that caused significant vimentin dispersion (Fig. 4c, FC > 1.4, *P* < 0.01), 6 of which were found to be MEK1/2 inhibitors (Fig. 4c; Fig. S4c, d). The unexpected enrichment of MEK1/2 pathway suggested MEK1/2 may be a novel regulator of vimentin.

We then applied a commonly used inhibitor (U0126) and activator (epidermal growth factor, EGF) of MEK1/2, respectively^39,40^, to examine their effect on vimentin and SCVs. U0126 treatment caused significant vimentin dispersion (Fig. 4e), while EGF led to vimentin aggregation (Fig. 4f). Immunofluorescence quantification further revealed that MEK1/2 activities are involved in scattering SCVs (Fig. 4g). MEK1/2 inhibitor dramatically decreased the percentage of SCV over dispersive SCV (Fig. 4g; Fig. S4e) as well as *Salmonella* replication (Fig. 4h). More importantly, either exogenously-expressed SopB-GFP or infection with *S*. Tm could augmented MEK1/2 phosphorylation (Fig. 4i). Compared to wild-type *S*. Tm infected cells, phosphorylation of MEK1/2 was decreased in cells infected by Δ*sopB* mutant (Fig. 4j), suggesting that SopB could modulate MEK1/2 activity. Therefore, these results showed that MEK1/2 is not only essential for vimentin rearrangement and SCVs maintenance, but also targeted by the *Salmonella* effector SopB (Fig. 4k).

### A SopB-Cdc42-MEK1/2-vimentin axis is required for SCV maintenance

**Fig. 5:**
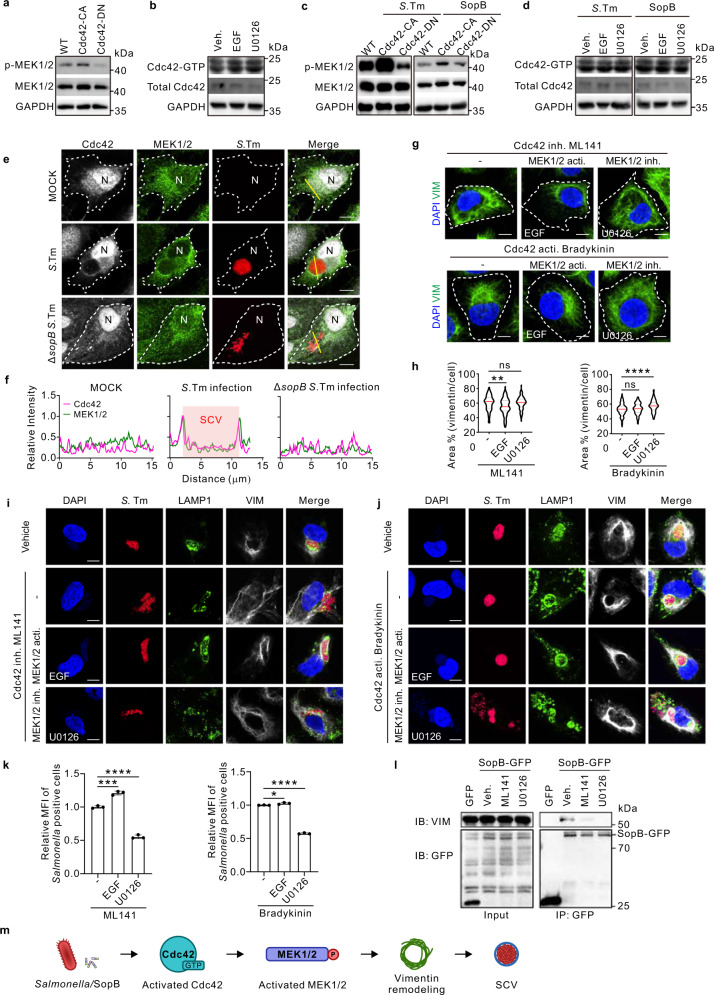
Cdc42 enhances MEK1/2 to regulate vimentin-mediated intracellular replication of *Salmonella*. **a** Western blotting analysis to detect p-MEK1/2 level in WT, constitutively-active (CA) or dominant-negative (DN) Cdc42 expressed cells. **b** Western blotting analysis to detect the active Cdc42 level in control, EGF and U0126 treated cells. **c** Western blotting analysis to detect p-MEK1/2 level in WT, Cdc42-CA, and Cdc42-DN expressed cells upon *Salmonella* infection (MOI = 20) or sopB-GFP transfection. **d** Western blotting analysis to detect the active Cdc42 level in control, EGF and U0126 treated cells upon *Salmonella* infection or sopB-GFP transfection. GAPDH serves as the loading control (**a**−**d**). **e** Immunofluorescence images of cells infected with *Salmonella* and Δ*sopB Salmonella* (MOI = 10) at 24 *hpi*. N indicated the nuclear position. **f** Relative fluorescence intensity profile of Cdc42 and MEK1/2 signaling based on the yellow line in (**e**). Pink square indicated the position of SCV. **g** Immunofluorescence images of cells treated with EGF or U0126, in the context of Cdc42 inhibition or activation, respectively. **h** Quantification of the relative vimentin area in (**g**) was measured. *n* = 20 views (60×/1.5 oil objective) from three independent experiments. **i**, **j** Immunofluorescence images of cells upon *Salmonella* infection (MOI = 10) at 24 *hpi*, treated with ML141 (**i**) or Bradykinin (**j**) in the context of MEK1/2 activation or inhibition, respectively. **k** Quantification of relative MFI values by FACS in cells upon *Salmonella* infection (MOI = 50) at 24 *hpi*, treated with the corresponding chemicals in (**i**, left panel) and (**j**, right panel), from three independent experiments. **l** Co-IP assay followed by Western blotting to test for association of SopB-GFP with vimentin upon Cdc42 or MEK1/2 inhibitor treatment. **m** Schematic diagram of *Salmonella*/SopB-Cdc42-MEK1/2 axis induced vimentin remodeling to surround SCV. White dash lines in (**e** and **g**) indicate the outline of cells. Assays were conducted three times independently with similar results (**a**–**e** and **i**). Scale bars, 10 μm (**e**, **g**, **i** and **j**). Data are represented as mean ± SD. Statistics (ns, *p* > 0.05; *p < 0.05; **p < 0.01; ***p < 0.001; ****p < 0.0001): one-way ANOVA with Dunnett’s analysis (**h**, **k**). Source data are provided as a Source Data file.

To dissect the functional relationships between MEK1/2 and Cdc42 in regulating vimentin/SCV maintenance, we overexpressed Cdc42-CA and Cdc42-DN mutations and found that MEK1/2 phosphorylation was activated by Cdc42-CA but inhibited by Cdc42-DN (Fig. 5a; Fig. S5a). Reciprocally, MEK1/2 activities did not affect the active level of Cdc42 (Fig. 5b), suggesting that Cdc42 is upstream of MEK1/2. Importantly, the unidirectional regulation of MEK1/2 activity by Cdc42 was recapitulated upon both *Salmonella* infection and SopB overexpression (Fig. 5c,d; Fig. S5b). MEK1/2, similar as Cdc42, are enriched around SCVs upon *Salmonella* infection, whereas it shows cytoplasmic diffused expression in Δ*sopB S*. Tm infection condition (Fig. 5e,f). These data indicated that Cdc42 is upstream of MEK1/2 in the regulation of SopB-dependent vimentin/SCV maintenance.

Indeed, MEK1/2 activation restored vimentin aggregation even when Cdc42 was inhibited, whereas simultaneous inhibition of MEK1/2 and Cdc42 was phenotypically consistent with Cdc42 inhibition alone (Fig. 5g, h). Furthermore, MEK1/2 inhibition suppressed the vimentin aggregation despite of Cdc42 activation, while simultaneous activating MEK1/2 and Cdc42 was phenotypically consistent with the Cdc42 activation effect alone (Fig. 5g, h). These permutational and combinational experiments suggested that MEK1/2 is a dominant downstream effector of Cdc42 for vimentin rearrangement. Consistently, upon *Salmonella* infection, increased dispersive SCVs by Cdc42 inhibition or Cdc42-DN were diminished by MEK1/2 activation (Fig. 5i; Fig. S5c, d). In contrast, MEK1/2 inhibition resulted in more dispersive SCVs in the presence of Cdc42 activation (Fig. 5j; Fig. S5e). Consistently, bacteria replication was elevated or suppressed by MEK1/2 activation or inhibitor, respectively, despite of Cdc42 inhibition or activation (Fig. 5k). It is important to note that the interaction between SopB and vimentin was disrupted by either inhibition of Cdc42 or MEK1/2, verified by co-IP assay (Fig. 5l). In conclusion, Cdc42 regulation of MEK1/2 activities is critical in vimentin-dependent maintenance of concrete SCVs (Fig. 5m).

### Trametinib, a clinically-approved MEK1/2 inhibitor, suppresses *Salmonella* infection both in vitro and in vivo

**Fig. 6:**
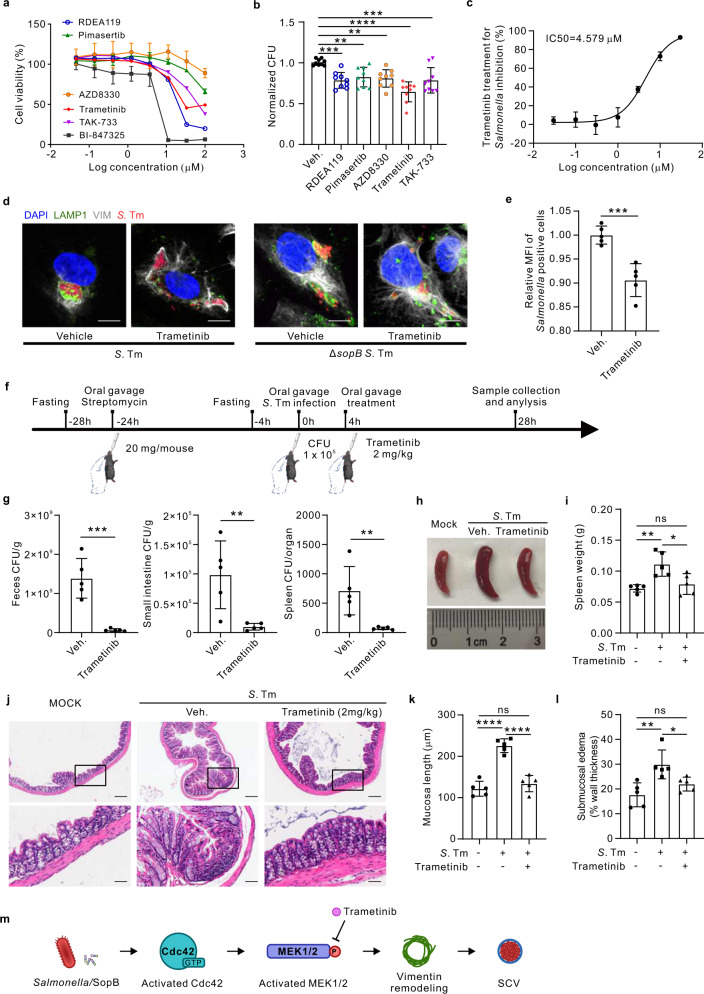
MEK1/2 inhibitors including the clinically-approved Trametinib show anti-infection effects of *Salmonella* in both cell and mouse model. **a** Quantification of the cell viability treated with the top six MEK1/2 inhibitors identified in the screen, from three independent experiments. **b** Quantification of the total intracellular bacterial colony forming unit (CFU) in cells infected with *Salmonella* (MOI = 10) and treated with five MEK1/2 inhibitors (1 µM) that showed no cytotoxic effects, at 24 *hpi* from three independent experiments. **c** Quantification of the *Salmonella* inhibition rate (MOI = 10) at 24 *hpi* with increasing concentrations of Trametinib. IC50 was calculated using nonlinear regression analysis from three independent experiments. **d** Immunofluorescence images of cells infected with *Salmonella* and Δ*sopB Salmonella* (MOI = 10) at 24 *hpi*, treated with Trametinib. Scale bars, 10 μm. **e** Quantification of relative MFI values by FACS in *Salmonella* infection cells treated with Trametinib at 24 *hpi*, from three independent experiments. **f** Schematic diagram of the oral gavage infections of *Salmonella* in mice enterocolitis model. **g** Bacterial loads in feces (left panel), small intestine (middle panel), and spleen (right panel) from *Salmonella* infected mice treated with or without Trametinib for 24 h. **h** Representative spleen of the mouse from each group was taken for photographic documentation. **i** Quantification of the total weight of spleen from uninfected and infected mice treated with or without Trametinib, respectively. **j** Representative images of H&E-stained colon sections illustrate pathological conditions of the mice. Scale bars, 100 μm in the upper images and 25 μm in the magnified images. **k**, **l** Quantification of the mucosal length (**k**) and submucosal edema (**l**) from uninfected and infected mice treated with or without Trametinib, respectively. **m** Schematic diagram of *Salmonella*/SopB-Cdc42-MEK1/2 axis induced vimentin remodeling to surround SCV, which was inhibited by MEK1/2 inhibitor Trametinib treatment. Data are represented as mean ± SD. *n* = 5 mice in (**g**, **i** and **k**, **l**). Statistics (ns, *p* > 0.05; *p < 0.05; **p < 0.01; ***p < 0.001; ****p < 0.0001): unpaired two-tailed Student’s *t*-test (**e**, **g**) or one-way ANOVA with Dunnett’s analysis (b, i, k and l). Source data are provided as a Source Data file.

Given the novel and critical role of MEK1/2 in maintaining concrete SCV, we set out to validate the six MEK1/2 inhibitors for their anti-bacterial activities (Fig. S6a). Five of all tested chemicals showed no cytotoxicity effect on cells until 10 μM (Fig. 6a), and all these five compounds effectively reduced *Salmonella* replication (Fig. 6b). The most potent compound was Trametinib (Fig. 6b), an FDA-approved drug for the treatment of metastatic melanoma^41^. Its cytotoxicity for longer treatment (e.g., 48 h) was excluded (Fig. S6b). Further analyses showed that Trametinib was fairly potent (IC50 = 4.579 μM, CC50 = 21.28 μM) in the inhibition of *Salmonella* infection (Fig. 6c; Fig. S6c). Time course studies verified that inhibition of Trametinib (1 μM) on MEK1/2 activities was effective from 3 h to 24 h post treatment, with total vimentin level unchanged (Fig. S6d). In addition, Trametinib (1 and 5 μM) was confirmed to cause a significant spread of vimentin (Fig. S6e, f). A similar effect of Trametinib on vimentin dispersion was also observed in L929 cells (Fig. S6g). Importantly, Trametinib treatment (1 μM, 24 h) significantly increased dispersive SCVs and reduced bacterial replication upon infection (Fig. 6d, e; Fig. S6h). Besides, SCVs dispersal was not further enhanced upon treatment with Trametinib in cells infected with Δ*sopB S*. Tm (Fig. 6d; Fig. S6h). To eliminate the direct impact of Trametinib on *S*. Tm, we cultured bacteria in the presence of Trametinib. The bacterial optical density showed that Trametinib did not affect the growth of bacteria per se (Fig. S6i). Moreover, we utilized these *S*. Tm to infect cells, which suggested that Trametinib-co-culture had no apparent effect on the bacterial infection efficiency (Fig. S6j). Together, these results suggested that MEK1/2 inhibition (e.g., by Trametinib) targeting vimentin/SCV can potently inhibit *Salmonella* infection in vitro.

To validate Trametinib efficacy in vivo, we employed the well-established streptomycin-treated mouse infection model, and applied 2 mg/kg of Trametinib treatment 4 h after infection (Fig. 6f). We determined the optimal oral gavage dose of *Salmonella* (10^5^ per animal) that caused enterocolitis (Fig. 6f) and bacteria burden post infection. Bacterial loads in the feces, small intestines and spleen were remarkably decreased by Trametinib treatment (Fig. 6g). Splenomegaly, typical for *Salmonella* infection, was also significantly reduced by Trametinib treatment (Fig. 6h, i), with drastic improvement of intestinal pathology (Fig. 6j), including mucosal thickening and submucosal edema reduction (Fig. 6k, l). Importantly, fluorescence imaging confirmed that Trametinib resulted in dispersed endogenous vimentin in mouse intestine (Fig. S6k). Cytokines detection by Luminex further showed significant reduction of inflammatory chemokines, tumor necrosis factors and interleukins upon Trametinib treatment (Fig. S7a–c). These results therefore showed that MEK inhibition by Trametinib effectively dampened *Salmonella* infection in vivo (Fig. 6m), and might be a promising therapeutic drug for enterocolitis.

**Fig. 7:**
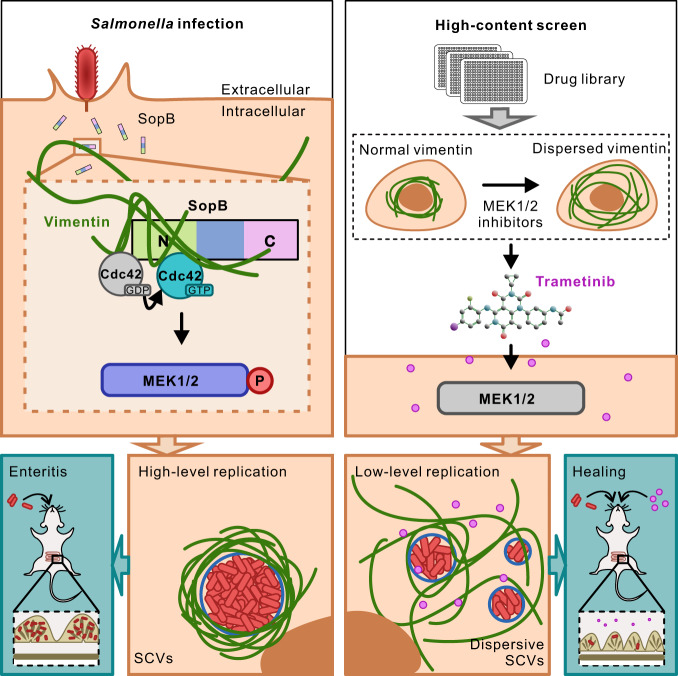
Schematic summary of *Salmonella* effector SopB reorganizes cytoskeletal vimentin for efficient infection. Bacterial SopB targets vimentin by its N terminal Cdc42-binding domain to maintain concrete replication vacuoles via activating Cdc42-MEK1/2 signaling axis. Trametinib, identified in the high-content screen, is a clinically approved drug inhibiting MEK1/2, which suppresses *Salmonella* infection both in vitro and in vivo.

To sum up, we unveiled a decisive mechanism contributing to *Salmonella* replication and a novel interplay between bacteria and host cytoskeleton. Post infection, *Salmonella* utilizes the N-terminal of T3SS effector SopB to bind and activate host Cdc42 GTPase, and subsequently promotes the phosphorylation of host MEK1/2. The activated MEK1/2 further induces the rearrangement of vimentin filaments to wrap around SCV, which facilitates to maintain the normal morphology of SCV and ensures the high-level replication of *Salmonella* (Fig. 7). Importantly, Trametinib, a clinically-approved drug from MEK1/2 inhibitors identified in the high-content screen, elicits dispersive SCVs and low-level replication of *Salmonella* both in vitro and in vivo (Fig. 7).

## Discussion

In this study, we report the spatio-temporal rearrangement of vimentin filaments and their crucial role during *Salmonella* infection. Importantly, we demonstrate that a vimentin-dependent remodeling of SCVs is essential to support the intracellular bacterial growth, and that vimentin remodeling is induced through a novel SopB-Cdc42-MEK1/2 signaling cascade. On these bases, we identify a clinically-approved drug, Trametinib, as a promising anti-*Salmonella* treatment in vivo. These results complement each other to provide integrated insights into the role of vimentin in regulating *Salmonella* infection. Moreover, this work encompassed the integration of most-advanced imaging techniques (e.g., live cell imaging, 3D-SIM super-resolution imaging, and high-content imaging), and provided a pipeline for identification (primary imaging-based screen) and validation (secondary infection screen) of novel anti-infection drug candidates.

Host microtubules and actin filaments have been extensively studied as critical drivers for the movement and positioning of SCVs^7,8,42–44^. Our findings on the function of vimentin in maintaining concrete SCV suggest that microtubules and actin filaments are not the only cytoskeleton elements involved. The roles of vimentin on SCVs uncovered in this study improve our understanding of the dynamic cytoskeletal forces involved in bacterial infection and how the bacteria control these forces to their benefit. The requirement for vimentin has also been indicated in other bacterial pathogens. For instance, bacteria-containing vacuole (BCV) formation during *Yersinia pseudotuberculosis* infection was significantly reduced in vimentin-depleted cells^24,45^, strengthening the hypothesis that bacteria may generally require vimentin to maintain the stability of their replication vacuoles. It was demonstrated previously that infection of vimentin-deficient cells with *Salmonella* resulted in a significant decrease in effector translocation^24^, indicating the potential effects on the entire bacterial infection process. This conclusion supports our findings of the role of vimentin on SCV formation and *Salmonella* replication. Whereas the possible effects on bacterial invasion might because of the different measurement methods and the infection cell models in two studies.

Among many effector proteins that manipulate SCV formation and maintenance, SopB is a major player regulating SCV formation at the early stage of infection^46^. In this study, we found SopB is essential for maintaining concrete SCV, and its deficiency fails to induce vimentin rearrangement in several different cell types (Fig. 2; Fig. S2). These results thus potentiated a new role of SopB which regulates SCV by recruiting vimentin at the later stage of infection (from 12 *hpi* onwards), suggesting that activities of this effector are even more dynamically coordinated than previously thought. Besides, SPI-1 effector SipB and SPI-2 effectors are inessential in regulating vimentin rearrangement, which were evidenced by Δ*sipB* and Δ*phoP* bacterial infection. However, whether other SPI-1 effectors, except for SopB and SipB, play important roles in this process needs to be further studied.

Most of the known functions of SopB, so far, were shown to depend on the phosphoinositide phosphatase activity in the C-terminus^47,48^. For instance, SopB mediates the recruitment of Rho GTPases (RhoB, RhoD, RhoH, and RhoJ) to the bacterial invasion sites^49^ and modulates SCV formation by maintaining high levels of phosphatidylinositol-three-phosphate (PtdIns(3)P) in the membrane of the SCV^50,51^. Nevertheless, our results demonstrated that the C-terminal phosphatase activity of SopB is dispensable for vimentin rearrangement (Fig. 3c), by infection of SopB^C460S^ expressed Δ*sopB S*. Tm. For the function of SopB N-terminus, previous work suggested that the binding of Cdc42 is related to bacterial entry and the localization of SopB to SCV^32,52^. Although SopB has been proposed to bind Cdc42^53^, there is no evidence that this interaction can activate Cdc42 and regulate the subsequent cytoskeleton remodeling. Data presented here suggest that SopB not only interacts with but also activates Cdc42 through its N-terminus, and more importantly, the N-terminal Cdc42 binding domain of SopB is indispensable for vimentin rearrangement, convinced by SopB^I49A^ mutant tool (Fig. 3c). These results expand a new functional layer in host cell processes that are targeted by SopB. Moreover, the interplay between vimentin and SopB identified here will open avenues for future mechanistic studies focusing on other Gram-negative intracellular bacterial infections.

As a crucial member of the Rho GTPase family required in cytoskeleton reorganization, cellular trafficking, and cell proliferation^54,55^, Cdc42 has been also repeatedly implicated in bacterial infection processes, where bacteria employ host Cdc42 to mediate their entry and/or their survival in their target cells^32,56^. Upon *Shigella* infection, a de novo actin polymerization and formation of an actin cocoon functioning as a gatekeeper for the cytosolic access of the pathogen are observed. Cdc42 is immediately recruited to the actin cocoon to promote its assembly, facilitating subsequent invasion steps for successful *Shigella* infection^56^. Our data, however, show that activated Cdc42 induces vimentin enrichment around replication vacuoles in *Salmonella* infection (Fig. 3d,e). The discrepancy suggested the possibility that Cdc42 may switch its preferred substrates between actin and vimentin, depending on specific pathogens, host cell types, and/or the time course of infection.

A previous study reported that the constitutively active Cdc42 mutant (Cdc42^V12^) can induce vimentin reorganization in HeLa cells^57^. They proposed that p21-activated kinase (PAK) and p70 S6 kinase (S6K) mediate the Cdc42-induced vimentin IF collapse^57^. This is mechanistically different from our results. By combining pharmacological inhibition, activity and functional assays, our data demonstrated that vimentin remodeling and bacterial replication, regardless of the active level of Cdc42 could be activated by MEK1/2 (Fig. 5). Thus, it is tempting to speculate that the intermediate regulatory kinase(s) between Cdc42 and vimentin may be dynamically regulated, and PAK&S6K and MEK1/2 are at least two schemes of the mediation. The activity of MEK1/2 is typically regulated by the rat sarcoma virus (Ras)/rapidly accelerated fibrosarcoma (Raf) signaling^58^. Whereas, we show here that activation of Cdc42 drives a non-canonical singling pathway that activates MEK1/2 (Fig. 5a-d). This finding is supported by another study in human neutrophils which reported a PI3K-Cdc42-PAK-MEK-ERK axis in regulating the immune-complex-induced apoptosis process^59^. Our results, therefore, may support a non-canonical but real mechanism of Ras/Raf-independent activation of MEK1/2.

Since MEK1/2 pathway is involved in the modulation of diverse cellular activities, including survival, differentiation, proliferation, and angiogenesis, chemicals inhibiting this cascade were mainly utilized to treat carcinoma^60^. Trametinib (GSK1120212), an oral drug and selective inhibitor of MEK1/2, has been approved by FDA for the treatment of metastatic melanoma^41^. There is no report correlating the subcellular distribution of vimentin with Trametinib according to our literature search. The rearrangement of endogenous vimentin observed in several cell lines (Fig. 6d; Fig. S6e-g) and mice intestinal cells (Fig. S6k) upon treatment with Trametinib, hence, validates the regulation of MEK1/2 on vimentin both in vitro and in vivo. Moreover, the bacterial infection assays in vitro and in vivo (Fig. 6b-i) also proved that Trametinib effectively dampened *Salmonella* infection. Trametinib has been used as a tool to inhibit MEK1/2 signaling, and shown to display anti-viral effect in HIV-1^61,62^, influenza A^63^ and SARS-CoV-2^64^ infections. On these bases, our work repurposed Trametinib as a possible anti-bacterial treatment for salmonellosis, which may help reduce the abuse of antibiotics, and expand the anti-infectious spectrum of Trametinib. Nevertheless, for the specific mechanism, we cannot evade a possibility that Trametinib may have additional effects on *Salmonella* infection bypassing bacterial SopB or host vimentin filaments, which is an interesting follow-up study in the near future.

## Methods

### Ethics statement

All animal experiments were conducted following institutional ethics requirements under the animal user permit (No. P2021014) approved by the Institute of Pasteur Animal Care Committee.

### Cell lines and bacteria strains

Human osteosarcoma U2OS cells, mouse macrophage cell line RAW 264.7, mouse embryonic fibroblasts cell line MEFs and mouse fibroblast L929 cells were maintained in Dulbecco’s Modified Eagle Medium (DMEM) (06-1055-57-1 A, Biological Industries, Kibbutz Beit-Haemek, Israel) supplemented with 10% fetal bovine serum (FBS, 10270-106, Gibco, Waltham, MA, United States), 100 U/ml Penicillin, and 100 µg/ml Streptomycin at 37 °C with 5% CO_2_. U2OS cells were gifts from Pekka Lappalainen (University of Helsinki), RAW 264.7 and MEFs were gifts from John Eriksson (University of Turku), and L929 cells were gifts from Li Yu (Tsinghua University). *Salmonella* Typhimurium (*S*. Tm; strain LT2) and Δ*sopB* mutant *Salmonella* Typhimurium (Δ*sopB S*. Tm; strain LT2) were kindly provided by Dr. Haihua Ruan (Tianjin University of Commerce, China). *Salmonella* Typhimurium (*S*. Tm; strain SL1344) was kindly provided by Dr. Feng Shao (National Institute of Biological Sciences, China). Unless indicated, strain LT2 (mcherry tagged) was used. The SopB-3xFLAG strain was constructed based on pSUB11 and the LamdaRED system. The Δ*sipB* and Δ*phoP* mutant *Salmonella* Typhimurium (Δ*sopB*, Δ*phoP S*. Tm; strain LT2) were constructed based on pSUB11 and the LamdaRED system. The SopB complemented strains were generated by introducing full length SopB, SopB mutant I49A or SopB mutant C460S into Δ*sopB* S. Tm using electroporation. Construction of Cdc42-CA and Cdc42-DN stable expression cell lines are briefly as follows: prepare lentivirus by transfection pCMV-VSV-G, pCMV delta8.9, and target gene plasmids (pCDH-CMV-Cdc42-CA-flag or pCDH-CMV-Cdc42-DN-flag) with the ratio of 1:2:4 into HEK293T cells. The lentivirus supernatants were collected 48 h after transfection, then concentrated in 8% PEG6000 for 24 h at 4 °C and centrifuged at 1500 g for 30 min at 4 °C. The pellets were resuspended in DMEM and aliquoted 250 μl per tube. 2 × 10^5^ U2OS cells were cultured in 6-well plate for 24 h and were then transduced with 250 μl lentivirus and 8 μg/ml polybrene for 12 h. Cells were then cultured for another 36 h. Fresh DMEM with 2 μg/ml Puromycin was added every 2 days for 10 days. Colonies were picked from the plate and expanded. Stable expression of target genes was verified in the corresponding cell lines by Western Blotting.

### Plasmids

Actin-mCherry was kindly provided by Dr. Pekka Lappalainen (University of Helsinki, Finland). Vector vimentin Y117L mutant (ULF) was kindly provided by Dr. John Eriksson (University of Turku, Finland). pCS2-EGFP-N1-SopB (Full-length, FL), pCS2-EGFP-N1-SopB (29-168 amino acid, SopB-N), and pCS2-EGFP-N1-SopB (168-561 amino acid, SopB-ΔN) were kindly provided by Dr. Haihua Ruan (Tianjin University of Commerce, China). Plasmids SopB used to construct complemented strain containing full length SopB was based on the pXG-10 vector. Primers used were designed as pXG-10-SopB-Forword 5' actgagcacatgcatgaggggaaatctgatggactataaagatgacgatgacaaacaaatacagagcttctatcactcagctt 3', pXG-10-SopB-reverse 5' tttgatgcctctagattaagatgtgattaatgaagaaatgccttttact3'. To obtain plasmids pXG-10-SopB^I49A^ and pXG-10-SopB^C460S^, we performed site-directed mutagenesis using pXG-10-SopB plasmid as template and the oligonucleotides I49A-1(5' tcgcccggaagctattgtcctgcgagaacccgg 3') and I49A-2 (5' cgcaggacaatagcttccgggcgagcgtcgg 3') or C460S-1 (5' cgcctggaattctaaaagcggcaaagatcgtacagg 3') and C460S-2 (5' tttgccgcttttagaattccaggcgggtaccgcg3') were used respectively. To generate vectors containing mCherry tag (PpagC-mCherry) and full-length vimentin with an N-terminal GFP tag in different expression vectors (plvx-GFP-vimentin, pcDNA-GFP-vimentin), a series of primers were designed as following: mCherry-forword 5'gaaggagatatacatatggtgagcaagggcgagga 3', mCherry-reverse 5'cgaaaacgccagctttcacttgtacagctcgtcca 3', Cdc42-forword 5' tgctctagaatggactacaaagacgat 3', Cdc42-reverse 5' gcgcctaggttagaatatacagcactt 3'. Plasmids pCXN2-Flag-Cdc42 V12 (CA) and pCXN2-Flag-Cdc42 N17 (DN) were gifts from Dr. Xueliang Zhu (University of Chinese Academy of Sciences). To produce plasmids that stably transfected into HEK293T cells through lentivirus, Cdc42 V12 (CA) and Cdc42 N17 (DN) were cloned into the pCDH recombinant lentiviral vectors (System Biosciences; Cat. #s CD510B-1). Primers used were designed as: pCDH-Cdc42-forword 5' tgctctagaatggactacaaagacgat 3', pCDH-Cdc42-reverse 5' gcgcctaggttagaatatacagcactt 3'.

### Construction of mCherry tagged bacteria strains

mCherry tagged *S*. Tm were transformed with PpagC-mCherry on which the promoter drives mCherry expression by electroporation. Briefly, bacteria were grown in Luria Broth (LB) to logarithmic phase, then wash and resuspend the bacteria in 10% glycerin, followed by incubation with PpagC-mCherry for 30 min. Thereafter the pulse 12.5 kV /cm (2.5 kV, 200 V, 25 MF) was applied, the electroporated products were further cultured for 1 h at 37°C and 220 rpm and then plated on the LB plates supplemented with 100 µg/ml ampicillin for further identification.

### *Salmonella* replication measurement in vitro

Cells were plated in 24-well plates with a density of 5 × 10^4^ cells per well. Before mcherry tagged *S*. Tm infection, bacteria were grown in LB medium for 12 h and transferred 1:33 to 2 ml fresh LB medium and grown for another 2 h to logarithmic phase. Bacteria were pelleted and resuspended in Phosphate Buffer Saline (PBS) buffer. Cells were infected with mcherry tagged *S*. Tm. Cells were then rinsed with PBS and replaced with DMEM containing 50 μg/ml gentamycin at 1 *hpi*. Cells were continued to culture for an additional 24 h. Cells for CFU assay were washed with PBS for 3 times and added with ddH_2_O for 15 min. Then the released mCherry-tagged bacteria were diluted and plated onto LB plates with 100 µg/ml ampicillin, and then incubated at 37 °C overnight for the measurement of CFU. Cells for Flow cytometry FACS analysis were digested by trypsin. Cells were then fixed with 4% PFA and resuspended in PBS by Flow cytometry (Aria III, BD, US; FACSDiva software, BD, US) using appropriate scatter gates and detection of mcherry fluorescence after excitation at 565 nm. The mean fluorescent intensity of the mcherry fluorescence was analyzed by Flowjo software (BD, US).

### The influence of Trametinib on the viability of *Salmonella*

MCherry tagged *S*. Tm were cultured with 1 µM Trametinib at 37 °C and 220 rpm in DMEM, and OD600 was measured at 0, 3, 6, 9, and 12 h, respectively. For invasion ability assay, mCherry tagged *S*. Tm were grown with 1 µM Trametinib in LB medium for 12 h, and transferred 1:33 to 2 ml fresh LB medium with 1 µM Trametinib, then grown for another 2 h to logarithmic phase. Bacteria were washed with PBS, and added to the cells at MOI 10. Cells were fixed at 1 *hpi*, and the percentage of infected cells was analyzed by imaging.

### Western blotting

Cells were washed with cold PBS and lysed in RIPA lysis buffer (Beyotime, P0013B, CN) supplied with protease and phosphatase inhibitors (Beyotime, P1045, CN) at 4 °C for 30 min. Cell debris was removed by centrifugation at 12,000 g for 20 min at 4 °C. Protein concentrations were determined by the Bradford assay kit (Beyotime, P0012, CN). Equal amounts of lysates were resolved by SDS-polyacrylamide gel electrophoresis and transferred to the PVDF membrane (Millipore, IPVH00010, DN). The membranes were blocked with 5% non-fat milk (BD, 8011939, US) in PBS-T buffer at room temperature (RT) for 30 min, and then incubated with primary antibodies overnight at 4 °C. After being washed with PBS for 10 min each 3 times, the membranes were incubated for 1 h with an appropriate horseradish peroxidase-conjugated secondary antibody (dilution 1:5000; #7076 V, CST, US). The following primary antibodies were used: vimentin rabbit monoclonal D21H3 antibody (dilution 1:1000; #5741, CST, US); MEK1/2 mouse monoclonal L38C12 antibody (dilution 1:1000; #4694, CST, US); phospho-MEK1/2 (Ser217/221) rabbit monoclonal 41G9 antibody (dilution 1:1000; #9154, CST, US); p44/42 MAPK (Erk1/2) (137F5) Rabbit mAb (dilution 1:1000; #4695, CST, US); Phospho-p44/42 MAPK (Erk1/2) (Thr202/Tyr204) (D13.14.4E) XP® Rabbit mAb (dilution 1:1000; #4370, CST, US); LAMP1 rabbit monoclonal C54H11 antibody (dilution 1:1000; #3243, CST, US); Polyclonal GroEL antibody (dilution 1:80,000; #G6532, Sigma-Aldrich, DN); Monoclonal Anti-FLAG (M2) antibody (dilution 1:1000; #F1804, Sigma-Aldrich, DN); Polyclonal Cdc42 Rabbit (dilution 1:1000; #10155-1-AP, Fisher Scientific, UK); Monoclonal Anti-Green Fluorescent Protein (GFP) antibody produced in mouse (dilution 1:1000; #G1546, Sigma-Aldrich, DN); GAPDH rabbit monoclonal antibody (dilution 1:5000; #G8795, Sigma-Aldrich, DN).

### Immunofluorescence microscopy

Cells were in a 24-well plate with a density of 1 × 10^4^ − 2 × 10^4^ cells per well on coverslips (631-0150, VWR, US). Cells were washed with PBS and fixed with 4% PFA for 15 min at RT, washed with PBS, and permeabilized with 0.1% Triton X-100 in PBS for 5 min. Cells were then blocked in 5% bovine serum albumin (BSA) (A23088, ABCONE, CN) in PBS for 30 min, and the following primary antibodies were used: vimentin rabbit monoclonal D21H3 antibody (dilution 1:100; #5741, CST, US); vimentin chicken polyclonal antibody (dilution 1:1000; ab24525, Abcam, UK); LAMP1 rabbit monoclonal D2D11antibody (dilution 1:100; #9091, CST, US). Both primary and secondary antibodies were applied to the cells and incubated at RT for 1 h sequentially. Alexa-conjugated phalloidin was added together with the secondary antibody solutions. After being washed three times with PBS, slices were mounted in DAPI fluoromount-G reagent (0100-20, SountherBiotech, Alabama) and subsequently the imaging data were obtained with Olympus SpinSR10 Ixplore spinning disk confocal microscope with UplanApo 60×/1.5 oil objective (Olympus Corporation, Tokyo, Japan) and super-resolution 3D-SIM OMX SR microscope (Cytiva, US). Images were analyzed with Image J (NIH, US) and Imaris 9.0 (Oxford Instruments, UK).

### Live cell imaging

Vimentin-GFP stable expression cells in 35 mm glass-bottom dishes (MatTek Corporation, P35G-1.5-14-C, US) in the density of 1 × 10^5^ cells per dish, followed by infection with mCherry tagged *S*. Tm at MOI of 10. Cells were rinsed with PBS and replaced with DMEM containing 50 μg/ml gentamycin after 1 h of infection. Cells were placed back in the incubator for an additional 2 h before live-cell imaging. The time-lapse images were acquired with Olympus cellSens Dimension system, consisting of an Olympus SpinSR10 Ixplore spinning disk confocal and a Yokogawa CSU-W1 confocal scanner. Appropriate filters, heated sample environment (37 °C), controlled 5% CO_2_ and UplanApo 100×/1.5 oil objective (Olympus Corporation, Tokyo, Japan) were used. The recording was set as every 36 min for 18 h and one focal plane was recorded for all live cell movies.

### Cell death assay

Cell death was measured by the CytoTox 96 Non-Radioactive Cytotoxicity Assay Kit (Promega, G1780, WI). Briefly, cells were seeded in 96-well plate in the density of 2 × 10^3^ cells per well. 50 µl of the cell culture supernatant was transferred to 96-well plate at indicated time point. For maximum LDH release control, lysis solutions were applied to the positive control wells. the cell supernatant was incubated with 50 µl of CytoTox 96 Reagent for 30 min at RT. The reaction was stopped with 50 µl of stop solution and the absorbance in each well at 490 nm was recorded with Synergy H1 Hybrid Multi-Mode Reader (BioTek, US). Cell death rates were calculated by comparing to the maximum LDH release control.

### Imaging-based drug screening

Actin-mCherry and vimentin-GFP double-labeled stable expression cells were seeded in 384-well plates. After cells reached 70%-80% confluence, 1694 inhibitors from Selleck chemicals library (US) were added at a dose of 1 µM, respectively. Three duplicates were performed for each compound. After 12 h of treatment, cells were washed three times with PBS and fixed with 4% PFA. The nuclei were counterstained with DAPI, and cells were imaged by Operetta CLS High-content Imaging and Analysis System (PerkinElmer, US).

### Inhibitors and activators treatment

Cells were infected with *S*. Tm at MOI of 10 for 1 h. Cells were then rinsed with PBS and replaced with DMEM containing 50 μg/ml gentamycin and one of the inhibitors or activators which are listed as follows: Cdc42 inhibitor, 5 µM ML141 (MedChem Express, HY-12755, CN); MEK1/2 inhibitors, 1 µM Trametinib (S2673), 1 µM RDEA119 (S1089), 1 µM Pimasertib (S1475), 1 µM AZD8330 (S2134), 1 µM TAK-733 (S2617), 1 µM BI-847325 (S7843), 10 µM U0126 (S1102) from Selleck chemicals (US); Cdc42 activator, 100 ng/ml Bradykinin (TargetMol, TP1277, CN); MEK activator, 100 ng/ml human EGF (Novoprotein, CA). Cells continued to incubate for an additional 24 h.

### Cell proliferation assay

Cell proliferation was measured by Cell Counting Kit-8 (Beyotime, C0040, CN). Briefly, cells were plated in the density of 2 × 10^3^ in 100 µl per well in 96-well culture plate and then incubated with 10 μl of CCK-8 regent at 37 °C for 0.5–4 h. Absorbance at 450 nm was then measured with Synergy H1 Hybrid Multi-Mode Reader (BioTek, US).

### Mass spectrometry

Infected cells were lysed with lysis buffer (50 mM pH8.0 Tris-HCl; 150 mM NaCl; 0.5% NP40; 1 mM EDTA; protease inhibitors (Beyotime, P1045, CN)) on ice for 10 min, then incubated with vimentin polyclonal antibody (ab137321, Abcam, UK) coated protein G dynabeads (10003D, Thermo Scientific, US) overnight for immunoprecipitation. Proteins pulled down by protein G dynabeads were washed by SDT buffer (4% SDS; 100 mM pH8.0 Tris-HCl; 1 mM DTT) for the Mass spectrum analyses by Q Exactive (Thermo Fisher, US). The qualitative appraisal target protein peptides were obtained by comparing *S*. Tm proteins with the Uniprot database. Differentially expressed eukaryotic protein co-immunoprecipitated with vimentin were identified. Gene Ontology terms (cellular component, biologic process, and molecular function) were considered significantly enriched. The mass spectrometry proteomics data have been deposited to the ProteomeXchange Consortium via the PRIDE partner repository with the dataset identifier PXD028785.

### Co-immunoprecipitation

Cells were transfected with pEGFP-N1, pCS2-EGFP-N1-SopB, pCS2-EGFP-N1-SopB-N, and pCS2-EGFP-N1-SopB-ΔN. For chemical treatment, ML141 or U0126 was added at 4 h post SopB-GFP transfection. After 16 h, cells were collected and washed with cold PBS and then lysed with the lysis buffer (50 mM pH8.0 Tris-HCl; 150 mM NaCl; 0.5% NP40; 1 mM EDTA; protease Inhibitors (Beyotime, P1045, CN)). Cell lysates were then centrifuged at 13,800 g for 10 min at 4 °C and the supernatant was collected. The protein concentration was determined by BCA Protein Assay Kit (Beyotime, P0010, CN). 50 µl supernatant was kept for the input detection and the rest of supernatant with same protein was incubated with anti-GFP beads (KT HEALTH, KTSM1301, CN) for 1 h at 4 °C. Beads were washed twice with lysis buffer and eluted with 100 μl SDS loading buffer at 95 °C for 10 min for further Western Blotting analysis.

### Cdc42 activity assay

Cdc42 activity assays were performed following the manufacturer’s protocol (CST, #8819). Briefly, cells were seeded in 6-well plate in the density of 4 × 10^5^ cells per well, washed in cold PBS and incubated for 5 min on ice in lysis buffer, and then centrifuged for 15 min at 16,000 g at 4 °C. The supernatant was incubated with GST-Rhotekin-RBD fusion protein, bound to glutathione-coupled Sepharose beads at 4 °C for 30 min. The beads and proteins bound to the fusion protein were washed three times with wash buffer, eluted in SDS buffer, and then analyzed by Western blotting using a monoclonal mouse antibody against human Cdc42.

### Mice infection

*S*. Tm (strain LT2) was grown overnight at 37 °C and then subcultured the next day in fresh LB at 37 °C to an OD600 0.9–1.0. Male 8 weeks old C57/B6j mice (*n* = 5 per group) were used in this study. Mice were housed in a SPF (specific pathogen free) facility with controlled temperature (18–22°C) and humidity (50–60%) and a 12-h dark/12-h light cycle. For streptomycin pretreatment, water and food were withdrawn 4 h before gavage. 24 h before infection 100 µl PBS containing 200 mg/ml of streptomycin was administered by oral gavage. Afterward, mice were supplied with water and food. For *S*. Tm infection, water and food were withdrawn 4 h before gavage, mice were inoculated with 1 × 10^5 ^*S*. Tm by oral gavage and then treated with 2 mg/kg body weight of Trametinib or the same amount of isotopic carrier (corn oil) at 4 *hpi* by oral gavage. Thereafter, drinking water and food were offered immediately. After 24 h, mice were euthanized and the feces, intestine, and spleen tissues were harvested and processed for CFU analysis. Colon was harvested and fixed in 4% PFA for tissue staining and immunofluorescence. Serums were collected for cytokine measurements by Luminex analyses using the Procartaplex Mouse 26-plex kit (Thermo Fisher, EPX260-26088-901, US). Samples were then analyzed on Bio-Plex 200 system (Bio-Rad, US).

### CFU analysis of mice tissues

The feces, small intestine, and spleen of infected mice were weighed before resuspending in 500 µl PBS. Tissues were homogenized in 70 HZ for 3 min (1 min each time for 3 times) with a tissue grinder (Scientz-48), homogenates were then serially diluted and 50 μl was plated onto LB plates with 100 µg/ml ampicillin. The plates were incubated at 37 °C and counted after overnight incubation. The CFU was normalized per gram weight for feces/small intestine or per organ for spleen.

### Tissue staining and immunofluorescence

Colons were fixed in 4% PFA for 24 h and washed with PBS, and then transferred to PBS containing 30% sucrose for 12 h. After that, colons were snap-frozen in Optimal Cutting Temperature (OCT) compound (Sakura, 4583) and were processed by a cryostat (Leica, CM1950) into 7 μm thick sections. Cut slices were collected using adhesion microscope slides (Citotest, 188105) and washed 3 times in PBS for 5 min. For Hematoxylin and Eosin (H&E) staining, tissue sections were stained with hematoxylin for 1 min and eosin for 30 sec, respectively. Mount coverslip onto the section on glass slide with neutral resins and subsequently the imaging data were obtained with Vectra3 with 20× objective (PerkinElmer, Vectra 3). Mucosa length and submucosal edema (% of the submucosa of the entire intestinal wall) were analyzed with Image J (NIH, US). For immunofluorescence staining, tissue slices were blocked in 5% BSA blocking solution for 1 h. The tissue slices were subsequently incubated with vimentin rabbit monoclonal D21H3 antibody (dilution 1:100; #5741, CST, US) in 5% BSA overnight at 4 °C, washed the next day with PBS and incubated with AF488 (Invitrogen, 710369) secondary antibody 1:1000 for 1 h. After three times washed with PBS, slices were mounted in DAPI fluoromount-G reagent (0100-20, SountherBiotech, Alabama) and subsequently the imaging data were obtained with Olympus SpinSR10 Ixplore spinning disk confocal microscope with UplanApo 60×/1.5 oil objective (Olympus Corporation, Tokyo, Japan). Images were analyzed with Imaris 9.0 (Oxford Instruments, UK).

### CellProfiler analysis of relative vimentin area

The analysis of the relative vimentin area was performed with CellProfiler Analyst software (CellProfiler 3.1.8; www.cellprofiler.org), which is capable of high-throughput imaging processing by automated computational analysis. We created a pipeline to identify the nucleus, vimentin, and actin of individual cells, respectively, (i) perform image preprocessing, (ii) segment and identify objects within the image by adjusting the threshold, (iii) and measure the area of vimentin and actin separately. We defined the whole cell area with the actin signal, and calculated the relative vimentin area through vimentin area divided by actin area.

### Quantification and statistical analysis

LAMP1 positive vacuolar containing >5 bacteria were defined as normal *Salmonella*-containing vacuole (SCV), <5 bacteria as dispersive SCV. For analysis of the vimentin intensity around SL1344, the average intensity of vimentin around the 1 μm width circle of SCVs was calculated by Imaris 9.0 (Oxford Instruments, UK). Statistical tests were selected based on appropriate assumptions concerning data distribution and variance characteristics. Statistical details of experiments can be found in the figure legends. Correlations and corresponding *p*-values were calculated by Pearson’s correlation test. ns *p* > 0.05; *p < 0.05; **p < 0.01; ***p < 0.001; ****p < 0.0001.

### Reporting summary

Further information on research design is available in the Nature Portfolio Reporting Summary linked to this article.

## Supplementary information

Supplementary Information

Description of Additional Supplementary Files

Supplementary Movie 1

Reporting Summary

## Data Availability

The authors declare that all data supporting the findings of the study are available in this article and its supplementary information files. The source data for Figures and Supplementary Figures generated in this study are provided in the Source Data file. The raw data for high-content imaging-based drug screening in this study is available on figshare (10.6084/m9.figshare.21621087). The mass spectrometry proteomics data have been deposited to the ProteomeXchange Consortium via the PRIDE partner repository with the dataset identifier PXD028785. Source data are provided with this paper.

## Source data

Source Data
