# Supplementary material for: Salmonella effector SopB reorganizes cytoskeletal vimentin to maintain replication vacuoles for efficient infection
Source: Nat Commun. 2023 Jan 30;14:478. doi: 10.1038/s41467-023-36123-w (PMC9885066; doi:10.1038/s41467-023-36123-w)
Supplement: Supplementary file 1 — Supplementary Information [file 41467_2023_36123_MOESM1_ESM.pdf]

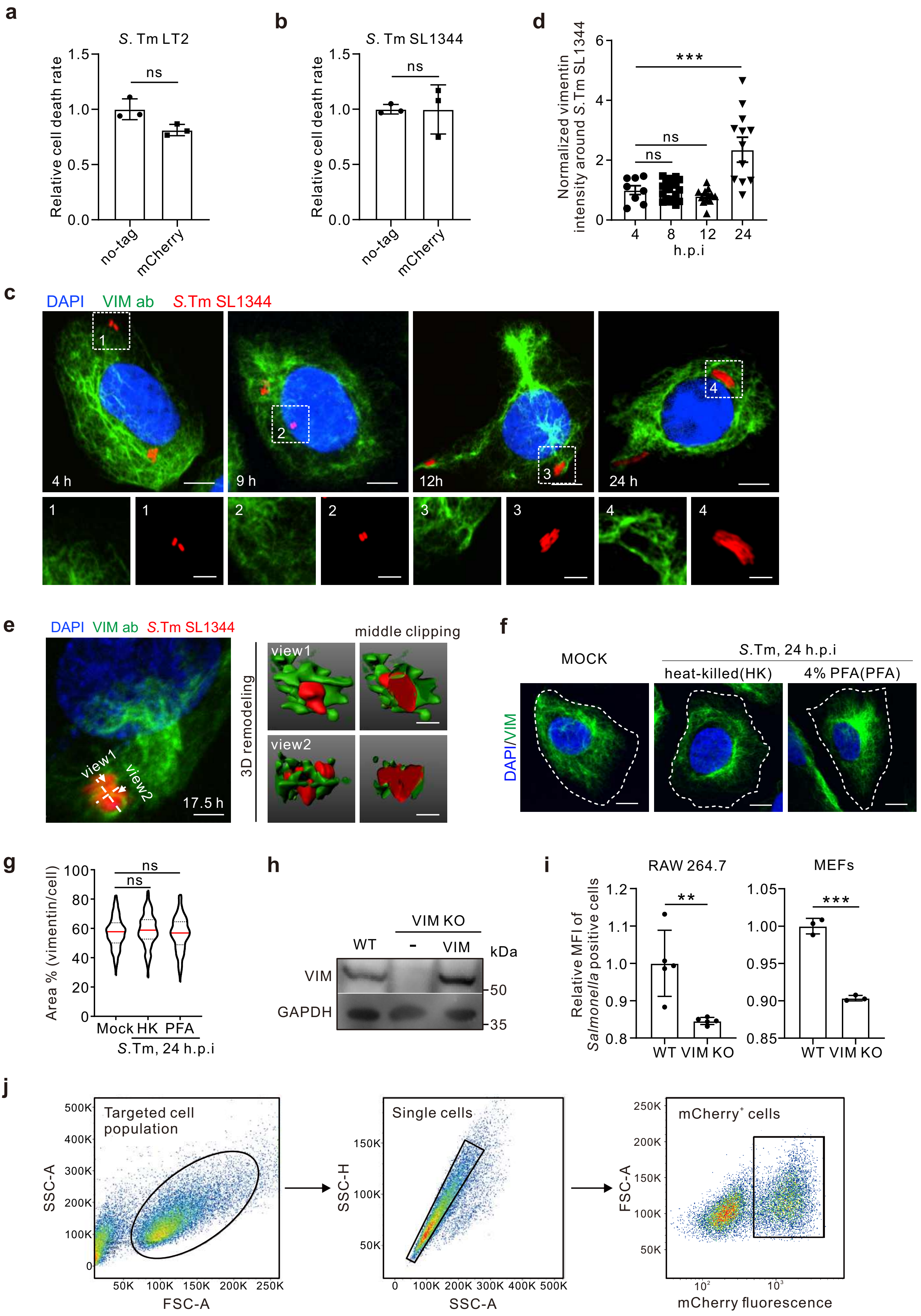
a
S. Tm LT2
Relative cell death rate
ns
no-tag
mCherry
b
S. Tm SL1344
Relative cell death rate
ns
no-tag
mCherry
d
Normalized vimentin intensity around S.Tm SL1344
***
ns
ns
h.p.i
c
DAPI
VIM ab
S.Tm SL1344
4 h
9 h
12h
24 h
e
DAPI VIM ab S.Tm SL1344
view1
view2
17.5 h
3D remodeling
middle clipping
f
MOCK
S.Tm, 24 h.p.i
heat-killed(HK)
4% PFA(PFA)
DAPI/VIM
g
Area % (vimentin/cell)
ns
ns
Mock HK PFA
S.Tm, 24 h.p.i
h
VIM KO
WT
-
VIM
kDa
VIM
GAPDH
50
35
i
RAW 264.7
MEFs
Relative MFI of Salmonella positive cells
**
***
WT VIM KO
j
Targeted cell population
SSC-A
FSC-A
Single cells
SSC-H
SSC-A
mCherry⁺ cells
FSC-A
mCherry fluorescence

Supplementary Figure 1. Cellular vimentin was rearranged upon different *Salmonella* strains infection, and vimentin is critical for *Salmonella* survival in different cell lines. a,**b**, Quantification of the normalized cell death rate in cells infected with the non-tagged and mCherry-tagged *S.* Tm (strain LT2; MOI=10) at 24 *hpi* (**a**) and non-tagged and mCherry-tagged *S.* Tm (strain SL1344; MOI=10) at 24 *hpi* (**b**), respectively, from three independent experiments. **c**, Immunofluorescence images of cells infected with the *S.* Tm (strain SL1344; MOI=10). Scale bars, 10 μm in the cell images and 2 μm in the magnified images. **d**, Quantification of the normalized vimentin-GFP intensity around *S.* Tm cluster (strain SL1344) in (**c**) was measured from three independent experiments. **e**, Representative image and the cross-section showing the cage-like structure of vimentin around *S.* Tm (strain SL1344; MOI=10) at 24 *hpi*. Scale bars, 10 μm in the cell image and 2 μm in the magnified images. Analysis was conducted three times independently with similar results. **f**, Immunofluorescence images of cells infected with the heat-killed (65°C for 10min) or 4% PFA (30min) treated *S.* Tm (strain LT2; MOI=10). White dash lines indicate the outline of the cells. Scale bars, 10 μm. **g**, Quantification of the relative vimentin area versus cell area in (**f**) were measured. n=20 views (60×/1.5 oil objective) from three independent experiments. **h**, Western blotting analysis to detect vimentin in WT, VIM KO and VIM-FL rescue U2OS cells. GAPDH serves as a loading control. **i**, Quantification of relative MFI values by FACS in WT and VIM KO RAW 264.7 or MEFs infected with mCherry-tagged *S.* Tm (MOI=50) at 24 *hpi*, respectively, from three independent experiments. **j**, Representative flow cytometric panels. The cells were gated to exclude debris and doublets. Mcherry-tagged bacteria infected cells were identified as mCherry^{+} cells. Data are represented as mean ± SD. Statistics (ns, $p > 0.05$; $^{**}p < 0.01$; $^{***}p < 0.001$): unpaired two-tailed Student's *t*-test (**a**, **b**, **i**) or one-way ANOVA with Dunnett's analysis (**d**, **g**). Source data are provided as a Source Data file.

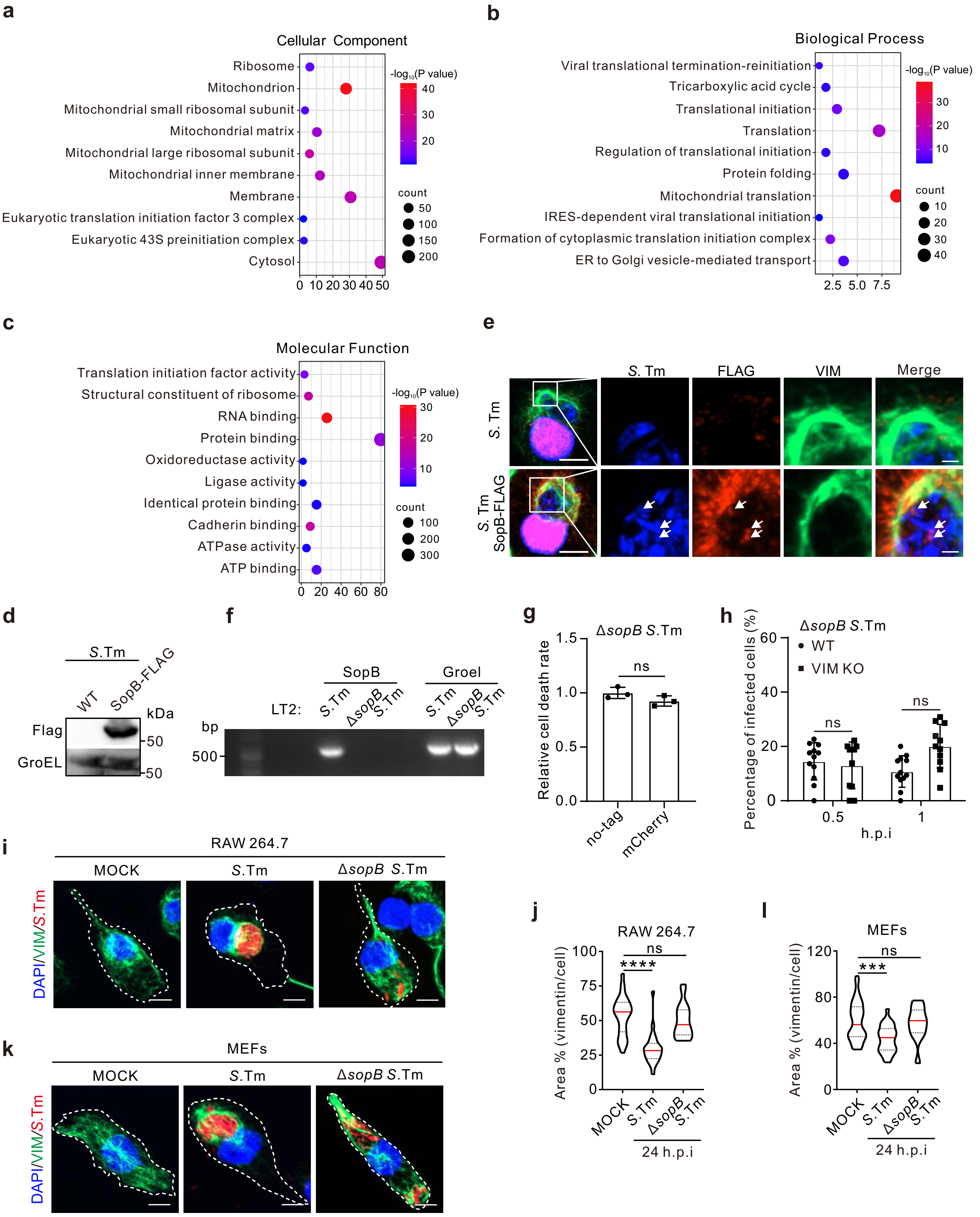

a
Cellular Component
Ribosome
Mitochondrion
Mitochondrial small ribosomal subunit
Mitochondrial matrix
Mitochondrial large ribosomal subunit
Mitochondrial inner membrane
Membrane
Eukaryotic translation initiation factor 3 complex
Eukaryotic 43S preinitiation complex
Cytosol
0 10 20 30 40 50
-log10(P value)
40
30
20
count
50
100
150
200
b
Biological Process
Viral translational termination-reinitiation
Tricarboxylic acid cycle
Translational initiation
Translation
Regulation of translational initiation
Protein folding
Mitochondrial translation
IRES-dependent viral translational initiation
Formation of cytoplasmic translation initiation complex
ER to Golgi vesicle-mediated transport
2.5 5.0 7.5
-log10(P value)
30
20
10
count
10
20
30
40
c
Molecular Function
Translation initiation factor activity
Structural constituent of ribosome
RNA binding
Protein binding
Oxidoreductase activity
Ligase activity
Identical protein binding
Cadherin binding
ATPase activity
ATP binding
0 20 40 60 80
-log10(P value)
30
20
10
count
100
200
300
e
S. Tm
FLAG
VIM
Merge
S. Tm
S. Tm SopB-FLAG
d
S.Tm
WT
SopB-FLAG
kDa
Flag
GroEL
50
50
f
SopB
Groel
LT2:
S.Tm
ΔsopB S.Tm
S.Tm
ΔsopB S.Tm
bp
500
g
ΔsopB S.Tm
Relative cell death rate
1.5
1.0
0.5
0.0
ns
no-tag
mCherry
h
ΔsopB S.Tm
WT
VIM KO
Percentage of infected cells (%)
60
40
20
0
ns
ns
0.5
1
h.p.i
i
RAW 264.7
MOCK
S.Tm
ΔsopB S.Tm
DAPI/VIM/S.Tm
k
MEFs
MOCK
S.Tm
ΔsopB S.Tm
DAPI/VIM/S.Tm
j
RAW 264.7
Area % (vimentin/cell)
100
75
50
25
0
ns
****
MOCK
S.Tm
ΔsopB S.Tm
24 h.p.i
l
MEFs
Area % (vimentin/cell)
120
80
40
0
ns
***
MOCK
S.Tm
ΔsopB S.Tm
24 h.p.i

Supplementary Figure 2. *Salmonella* infection induced variation of vimentin interacting components from host cells, and SopB is critical for vimentin rearrangement in different cell lines. **a-c**, GO analysis of all identified eukaryotic proteins co-immunoprecipited with vimentin by mass spectrometry. Top 10 significant enrichments of cellular component (**a**), biologic process (**b**) and molecular function (**c**) were shown in the bubble chart, respectively. **d**, Western blotting analysis to detect the FLAG-tagged SopB in *S.* Tm. GroEL serves as a loading control. **e**, Immunofluorescence images of cells infected with non-tagged *S.* Tm or SopB-FLAG *S.* Tm (MOI=10) at 24 *hpi*. White arrows indicate SopB signals within bacteria. Scale bars, 10 μm in the cell images and 3 μm in the magnified images. **f**, Agarose gels analysis to detect the PCR products amplifying sopB from genomic DNA of *S.* Tm or Δ*sopB S.* Tm. **g**, Quantification of the normalized cell death rate in cells infected with the non-tagged or mCherry-tagged Δ*sopB S.* Tm (MOI=10) at 24 *hpi*, from three independent experiments. **h**, Percentage of cells in WT and VIM KO background infected with the Δ*sopB S.* Tm (MOI=10) at 0.5 and 1 *hpi*, respectively, from three independent experiments. **i**, Immunofluorescence images of RAW 264.7 infected with *S.* Tm or Δ*sopB S.* Tm (MOI=10) at 24 *hpi*. Scale bars, 10 μm. **j**, Quantification of the relative vimentin area versus cell area in (**i**) was measured. **k**, Immunofluorescence images of MEFs infected with *S.* Tm or Δ*sopB S.* Tm (MOI=10) at 24 *hpi*. Scale bars, 10 μm. **l**, Quantification of the relative vimentin area versus cell area in (**k**) was measured. n=20 views (60×/1.5 oil objective) from three independent experiments in (**j**, **l**). White dash lines in (**i**, **k**) indicate the outline of the cells. Assays were conducted three times independently with similar results (**d-f**). Data are represented as mean ± SD. Statistics (ns, $p > 0.05$; ***$p < 0.001$; ****$p < 0.0001$): unpaired two-tailed Student's *t*-test (**g-h**), one-way ANOVA with Dunnett's analysis (**j**, **l**), or EASE score (**a-c**). Source data are provided as a Source Data file.

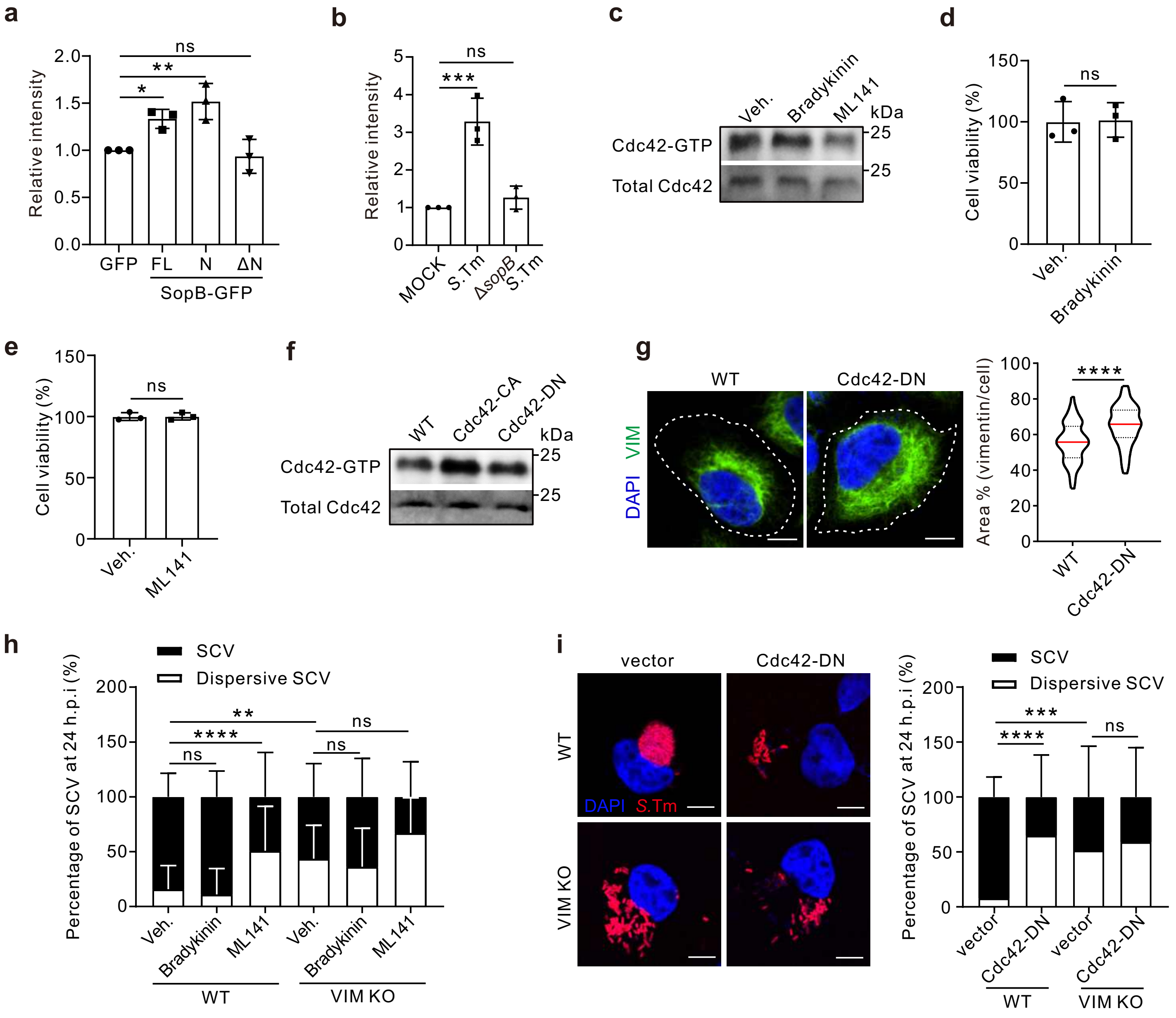

Supplementary Figure 3. Cdc42 is essential for vimentin rearrangement and SCV maintenance. a, Quantification of Cdc42-GTP relative to total Cdc42 upon SopB constructs transfection corresponding to Fig. 3f, from three independent experiments. **b**, Quantification of Cdc42-GTP relative to total Cdc42 upon infection with WT and Δ*sopB S.* Tm (MOI=10) corresponding to Fig. 3g, from three independent experiments. **c**, Western blotting analysis to detect the GTP-bound active level of Cdc42 upon treatment with its activator bradykinin (100 ng/ml) and inhibitor ML141 (5 μM), respectively. **d**,**e**, Quantification of the cell viability treated with Bradykinin (**d**) and ML141 (**e**) for 24 h was measured respectively. **f**, Western blotting analysis to detect the GTP-bound active level of Cdc42 in WT, constitutively active Cdc42 (Cdc42-CA) and dominant negative Cdc42 (Cdc42-DN) cells, respectively, from three independent experiments. **g**, Immunofluorescence images of WT and Cdc42-DN stalely expressed cells. White dash lines indicate the outline of the cells. Scale bars, 10 μm. Right panel shows the quantification of the relative vimentin area versus cell area. **h**, Quantification of the percentage of SCV versus dispersive SCV corresponding to Fig. 3j. **i**, Immunofluorescence images of WT and Cdc42-DN stably expressed WT and VIM KO cells infected with the *S.* Tm (MOI=10) at 24 *hpi*. Scale bars, 10 μm. Right panel shows the quantification of the percentage of SCV versus dispersive SCV. n=20 views (60×/1.5 oil objective) from three independent experiments in (**g-i**). Assays were conducted three times independently with similar results (**c**, **f**). Data are represented as mean ± SD. Statistics (ns, $p > 0.05$; $*p < 0.05$; $**p < 0.01$; $***p < 0.001$; $****p < 0.0001$): unpaired two-tailed Student's *t*-test (**d**, **e**, **g** right panel); one-way ANOVA with Dunnett's analysis (**a**, **b**) or two-way ANOVA with Sidak's analysis (**h, i** right panel). Source data are provided as a Source Data file.

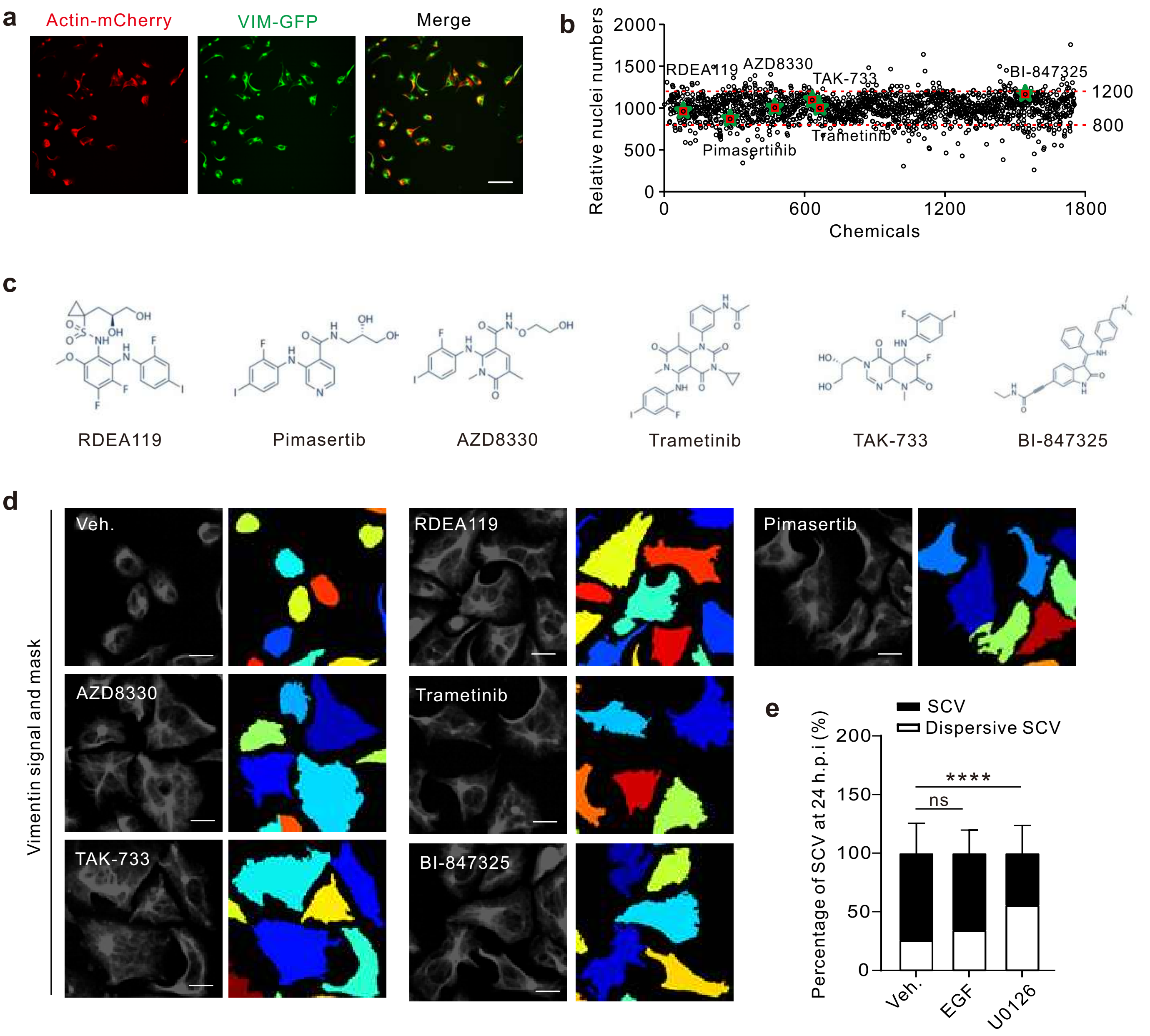

Supplementary Figure 4. Candidate chemicals from high-content screens showed no obvious cell cytotoxicity. a, Immunofluorescence images of VIM-GFP and Actin-mCherry stably expressing cell line. Scale bars, 100 μm. **b**, Quantification of the relative nuclei numbers of chemically treated cells. The top six candidates in MEK1/2 pathway are highlighted as the green star. **c**, The chemical structures of the top six MEK1/2 inhibitors from the screen. **d**, Representative images of vimentin treated with the top six MEK1/2 inhibitors, respectively. Scale bars, 20 μm. **e**, Quantification of the percentage of SCV versus dispersive SCV corresponding to Fig. 4g. n=20 views (60×/1.5 oil objective) from three independent experiments. Assays were conducted three times independently with similar results (**a**, **d**). Data are represented as mean ± SD. Statistics (ns, $p > 0.05$; ****$p < 0.0001$): two-way ANOVA with Sidak's analysis (**e**). Source data are provided as a Source Data file.

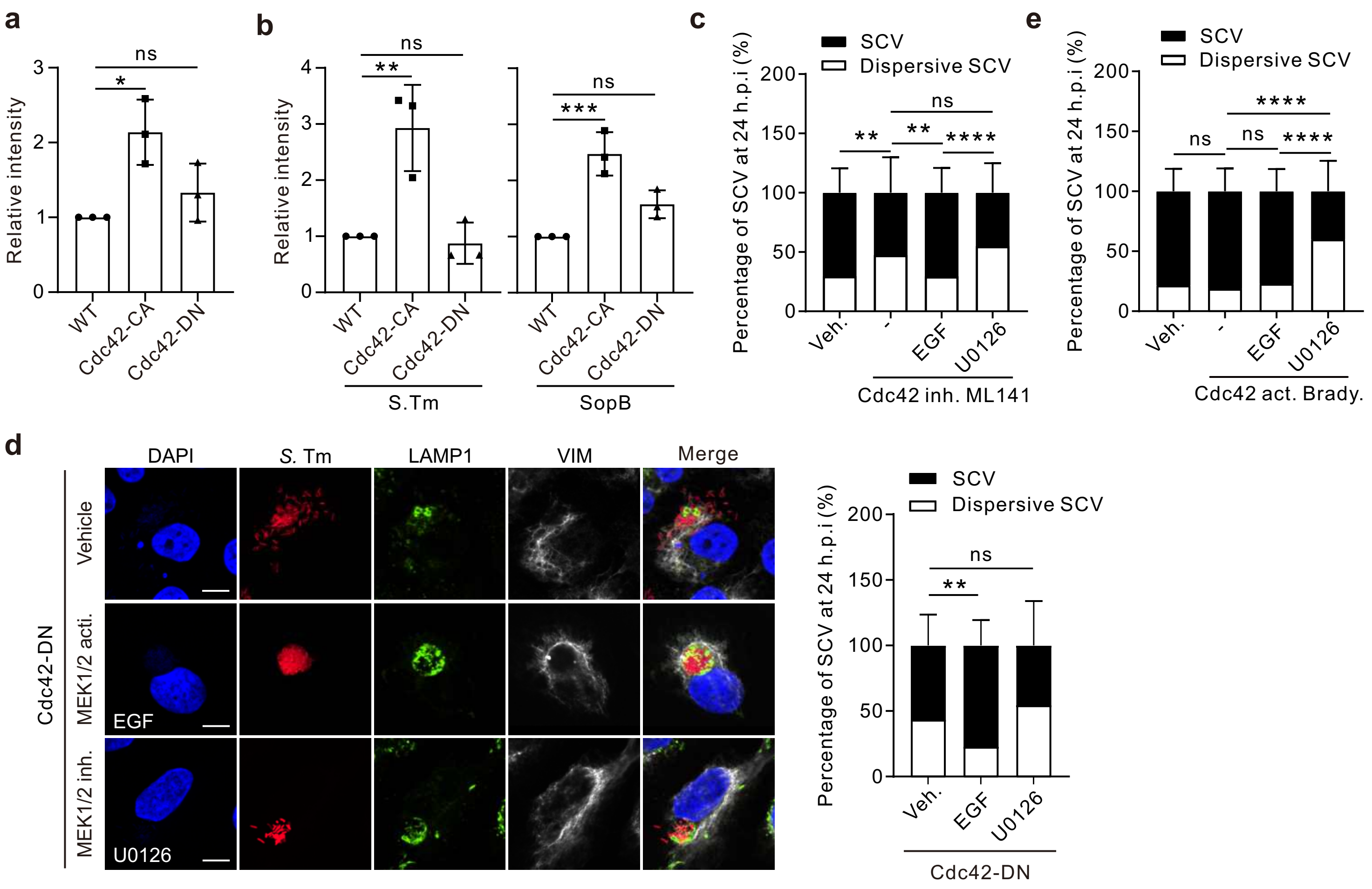

Supplementary Figure 5. Verification of MEK1/2-Cdc42 regulation. a,**b**, Quantification of p-MEK1/2 relative to total MEK1/2 corresponding to Fig. 5a and 5c, respectively, from three independent experiments. **c**, Quantification of the percentage of SCV versus dispersive SCV corresponding to Fig. 5i. **d**, Immunofluorescence images of Cdc42-DN stably expressed cells infected with *S.* Tm (MOI=10) at 24 *hpi* with or without treatment of MEK1/2 activator EGF (100 ng/ml) or inhibitor U0126 (10 μM), respectively. Scale bars, 10 μm. Quantification of the percentage of SCV versus dispersive SCV is shown in the right panel. **e**, Quantification of the percentage of SCV versus dispersive SCV corresponding to Fig. 5j. n=20 views (60×/1.5 oil objective) from three independent experiments in (**c**-**e**). Data are representative as mean ± SD. Statistics (ns, $p > 0.05$; $*p < 0.05$; $**p < 0.01$; $***p < 0.001$; $****p < 0.0001$): one-way ANOVA with Dunnett's analysis (**a**, **b**) or two-way ANOVA with Sidak's analysis (**c**-**e**). Source data are provided as a Source Data file.

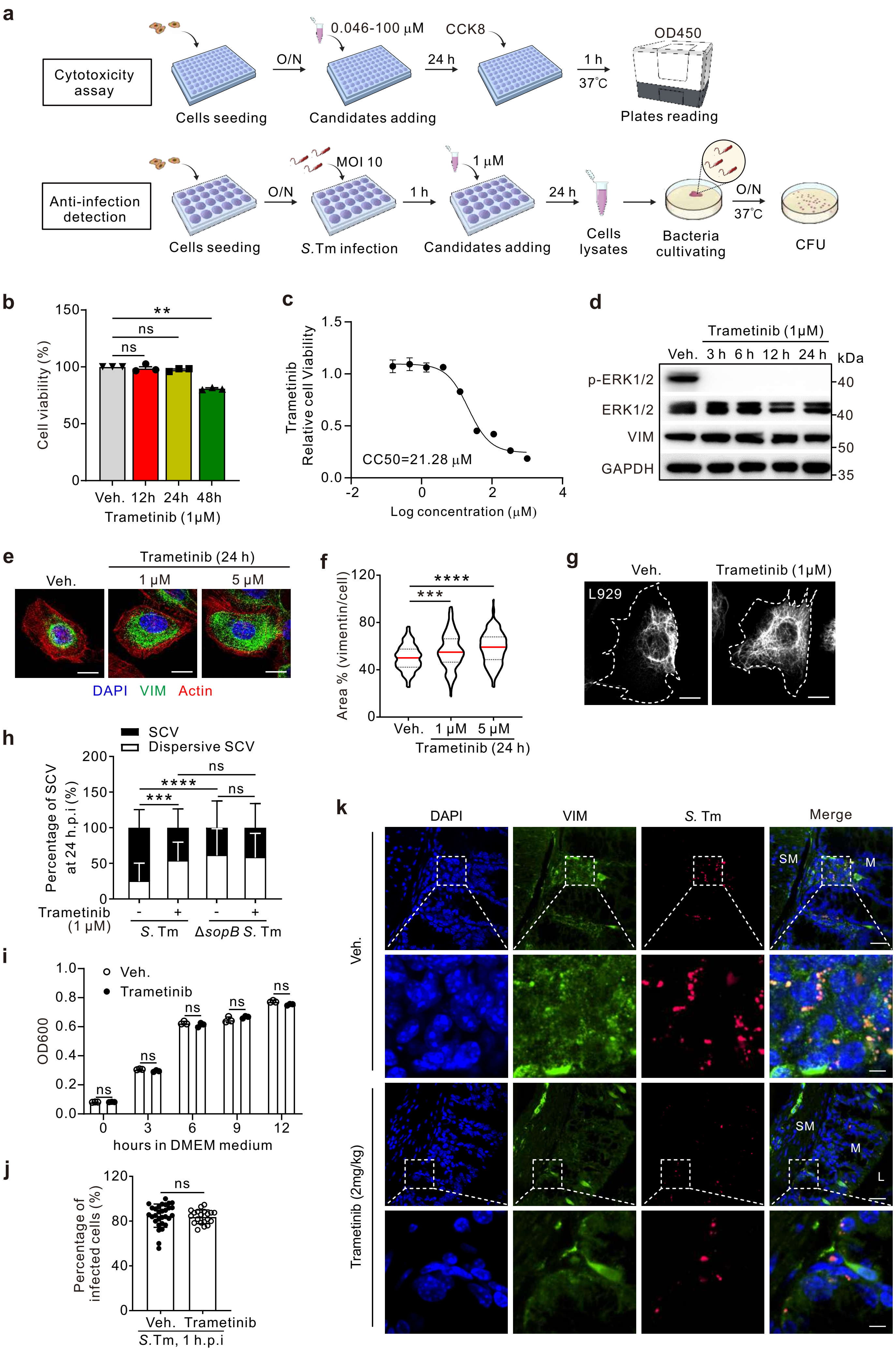
a
Cytotoxicity assay
Cells seeding
O/N
0.046-100 μM
Candidates adding
24 h
CCK8
1 h
37°C
OD450
Plates reading
Anti-infection detection
Cells seeding
O/N
MOI 10
S.Tm infection
1 h
1 μM
Candidates adding
24 h
Cells lysates
Bacteria cultivating
O/N
37°C
CFU
b
Cell viability (%)
**
ns
ns
Veh. 12h 24h 48h
Trametinib (1μM)
c
Trametinib Relative cell Viability
CC50=21.28 μM
Log concentration (μM)
d
Trametinib (1μM)
Veh. 3 h 6 h 12 h 24 h
kDa
p-ERK1/2
ERK1/2
VIM
GAPDH
40
40
50
35
e
Trametinib (24 h)
Veh. 1 μM 5 μM
DAPI VIM Actin
f
Area % (vimentin/cell)
****
***
Veh. 1 μM 5 μM
Trametinib (24 h)
g
Veh. Trametinib (1μM)
L929
h
SCV
Dispersive SCV
Percentage of SCV at 24 h.p.i (%)
ns
****
***
ns
Trametinib (1 μM)
- + - +
S. Tm
ΔsopB S. Tm
i
Veh.
Trametinib
OD600
ns
hours in DMEM medium
j
Percentage of infected cells (%)
ns
Veh. Trametinib
S.Tm, 1 h.p.i
k
DAPI
VIM
S. Tm
Merge
Veh.
Trametinib (2mg/kg)
SM
M
L

Supplementary Figure S6. Verification of Trametinib effects. a, Schematic diagram of the secondary screen with cytotoxicity and anti-infection detection as a readout for the top six MEK1/2 inhibitors from the primary imaging-based screen. **b**, Quantification of the cell viability of cells that treated with Trametinib (1μM) for different time points, from three independent experiments. **c**, Quantification of the relative cell viability in increasing concentrations of Trametinib indicting the CC50 from three independent experiments. **d**, Western blotting verified the effects of Trametinib on the inhibition of MEK1/2 activity. GAPDH serves as a loading control. Assays were conducted three times independently with similar results. **e**, Immunofluorescence images of vimentin in cells treated with 1 μM and 5 μM Trametinib for 24 h, respectively. Scale bars, 10 μm. **f**, Quantification of the relative vimentin area versus cell area in (e) was measured. n=30 views (60×/1.5 oil objective) from three independent experiments. **g**, Immunofluorescence images of L929 cells treated without or with Trametinib (1μM) for 24h. Scale bars, 10 μm. **h**, Quantification of the percentage of SCV versus dispersive SCV corresponding to Fig. 6d. n=20 views (60×/1.5 oil objective) from three independent experiments. **i**, Growth rates of *S.* Tm or Trametinib-treated *S.* Tm were quantified by OD600 measurement at 0, 3, 6, 9, 12 *hpi*, from three independent experiments. **j**, Percentage of *S.* Tm or Trametinib-treated *S.* Tm (MOI=10) infected cells was analyzed at 24*hpi*. n=20 views (60×/1.5 oil objective) from three independent experiments. **k**, Immunofluorescence staining of vimentin in the colon tissue slices of infected mice treated with or without Trametinib (2mg/kg). Scale bars, 30 μm in the upper images and 10 μm in the magnified images. Mucosal (M); submucosal (SM); lumen (L) (n=5 mice). Data are representative as mean ± SD. Statistics (ns, $p > 0.05$;**$p < 0.01$; ***$p < 0.001$; ****$p <0.0001$): unpaired two-tailed Student's *t*-test (**j**), one-way ANOVA with Dunnett's analysis (**b**, **f**) or two-way ANOVA with Sidak's analysis (**h**, **i**). Source data are provided as a Source Data file.

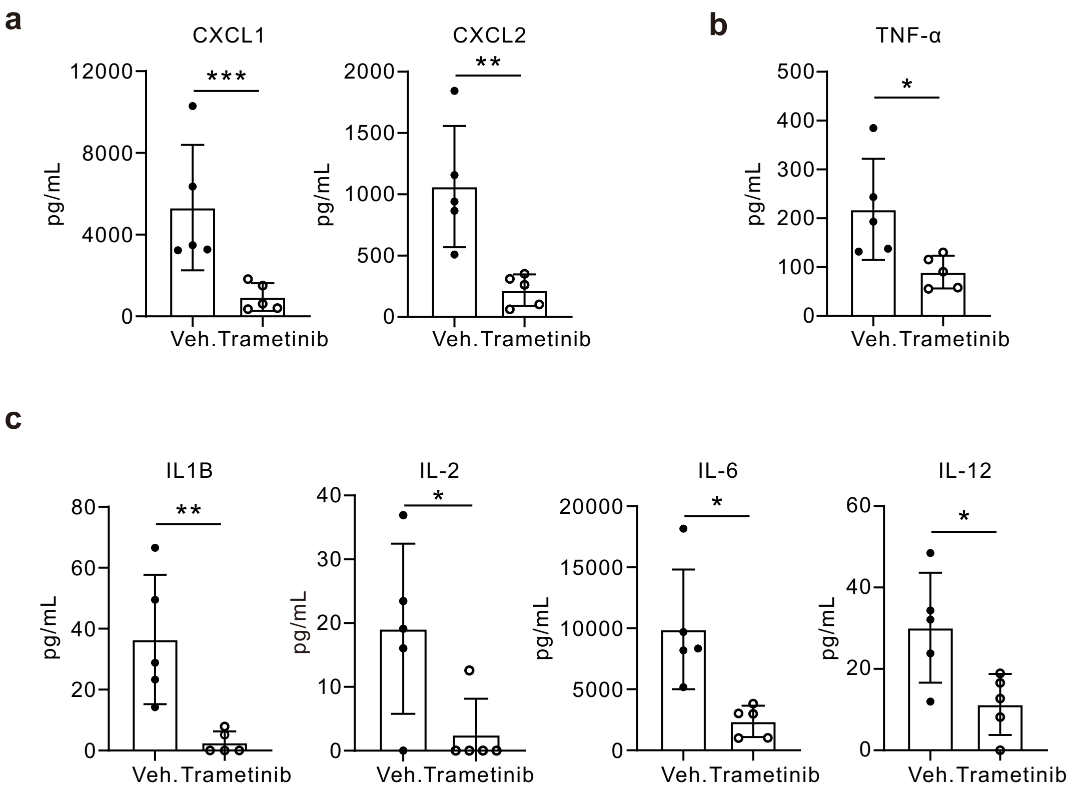

Supplementary Figure 7. The *Salmonella*-induced inflammatory response in mice infected model. **a-c**, Chemokines (CXCL1; CXCL2) (**a**), Tumor necrosis factor (TNFα) (**b**), and Interleukins (IL1B; IL-2; IL-6; IL-12) (**c**) from *S.* Tm infected mice model treated with or without Trametinib (2 mg/kg) (n=5). Data are representative as mean ± SD. Statistics (*$p < 0.05$; **$p < 0.01$; ***$p < 0.001$): unpaired two-tailed Student's t-test (**a-c**). Source data are provided as a Source Data file.
