# Supplementary material for: Salmonella effector SopB reorganizes cytoskeletal vimentin to maintain replication vacuoles for efficient infection
Source: Nat Commun. 2023 Jan 30;14:478. doi: 10.1038/s41467-023-36123-w (PMC9885066; doi:10.1038/s41467-023-36123-w)
Supplement: Supplementary file 2 — Description of Additional Supplementary Files [file 41467_2023_36123_MOESM2_ESM.pdf]

Description of Additional Supplementary Files

File Name: Supplementary Movie 1
Description: Time-lapse movie of vimentin-GFP expressing cells infected with the mCherry tagged S. Tm. The recording time interval is 0.6 h / frame. The display rate is 3 frames / second. Scale bar, 20 µm.
