# Supplementary material for: Salmonella effector SopB reorganizes cytoskeletal vimentin to maintain replication vacuoles for efficient infection
Source: Nat Commun. 2023 Jan 30;14:478. doi: 10.1038/s41467-023-36123-w (PMC9885066; doi:10.1038/s41467-023-36123-w)
Supplement: Supplementary file 4 — Reporting Summary [file 41467_2023_36123_MOESM4_ESM.pdf]

nature portfolio

Corresponding author(s): Yaming Jiu
Last updated by author(s): Dec 1, 2022

Reporting Summary

Nature Portfolio wishes to improve the reproducibility of the work that we publish. This form provides structure for consistency and transparency in reporting. For further information on Nature Portfolio policies, see our Editorial Policies and the Editorial Policy Checklist.

Statistics

For all statistical analyses, confirm that the following items are present in the figure legend, table legend, main text, or Methods section.

n/a	Confirmed	
☐	☒	The exact sample size (*n*) for each experimental group/condition, given as a discrete number and unit of measurement
☐	☒	A statement on whether measurements were taken from distinct samples or whether the same sample was measured repeatedly
☐	☒	The statistical test(s) used AND whether they are one- or two-sided<br>*Only common tests should be described solely by name; describe more complex techniques in the Methods section.*
☐	☒	A description of all covariates tested
☐	☒	A description of any assumptions or corrections, such as tests of normality and adjustment for multiple comparisons
☐	☒	A full description of the statistical parameters including central tendency (e.g. means) or other basic estimates (e.g. regression coefficient) AND variation (e.g. standard deviation) or associated estimates of uncertainty (e.g. confidence intervals)
☐	☒	For null hypothesis testing, the test statistic (e.g. *F*, *t*, *r*) with confidence intervals, effect sizes, degrees of freedom and *P* value noted<br>*Give P values as exact values whenever suitable.*
☒	☐	For Bayesian analysis, information on the choice of priors and Markov chain Monte Carlo settings
☒	☐	For hierarchical and complex designs, identification of the appropriate level for tests and full reporting of outcomes
☐	☒	Estimates of effect sizes (e.g. Cohen's *d*, Pearson's *r*), indicating how they were calculated

Our web collection on statistics for biologists contains articles on many of the points above.

Software and code

Policy information about availability of computer code

Data collection	Images were collected by the Olympus spinsr Nipkow double turntable system controlled by cellSens Dimension software;<br>MFI (Mean Fluorescence Intensity) was collected by the BD Celesta controlled by FACSDiva Software;<br>Tissue slice imaging was collected by PerkinElmer Vectra 3 automated quantitative pathology imaging system.
Data analysis	3D reconstruction and movie acquisition were done by imaris 9.0 software (https://imaris.oxinst.cn/microscopy-imaging-software-free-trial);<br>Relative vimentin area was analyzed using Cellprofilier 3.1.8 (https://cellprofiler.org);<br>Analysis was performed using the Graphpad Prism 8.0.2 (https://www.graphpad.com/scientific-software/prism/)<br>Line profile was analyzed using Image J/Fiji 2.9.0(https://imagej.net/software/fiji);<br>MFI was analyzed using FlowJo 10.5 (https://info.flowjo.com/flowjo-10.5-release).

For manuscripts utilizing custom algorithms or software that are central to the research but not yet described in published literature, software must be made available to editors and reviewers. We strongly encourage code deposition in a community repository (e.g. GitHub). See the Nature Portfolio guidelines for submitting code & software for further information.

Data

Policy information about availability of data

All manuscripts must include a data availability statement. This statement should provide the following information, where applicable:

- Accession codes, unique identifiers, or web links for publicly available datasets
- A description of any restrictions on data availability
- For clinical datasets or third party data, please ensure that the statement adheres to our policy

The authors declare that all data supporting the findings of the study are available in this article and its supplementary information files. The source data for Figures and Supplementary Figures generated in this study are provided in the Source Data file. The raw data for high-content imaging-based drug screening in this study is available on figshare (https://doi.org/10.6084/m9.figshare.21621087). The mass spectrometry proteomics data have been deposited to the ProteomeXchange Consortium via the PRIDE partner repository with the dataset identifier PXD028785. Source data are provided with this paper.

Human research participants

Policy information about studies involving human research participants and Sex and Gender in Research.

Reporting on sex and gender	N/A
Population characteristics	N/A
Recruitment	N/A
Ethics oversight	N/A

Note that full information on the approval of the study protocol must also be provided in the manuscript.

Field-specific reporting

Please select the one below that is the best fit for your research. If you are not sure, read the appropriate sections before making your selection.

☒ Life sciences ☐ Behavioural & social sciences ☐ Ecological, evolutionary & environmental sciences

For a reference copy of the document with all sections, see nature.com/documents/nr-reporting-summary-flat.pdf

Life sciences study design

All studies must disclose on these points even when the disclosure is negative.

Sample size	For cellular experiments, 20 or 30 views (60×/1.5 oil objective) per group were analyzed. As for infection assays, only one infected cell in some views. To ensure there were at least 30 cells per group, 20 or 30 views per group were analyzed. Sample size estimation was carried out using prior data for expected sample means and standard deviations generated in murine Salmonella typhimurium infection models.
Data exclusions	No data were excluded from the analyses.
Replication	Results of experiments were reliably reproduced at least 3 times.
Randomization	For mice experiments, animals in each cage were randomly assigned to either vehicle or treatment groups. For in vitro experiments using cell line allocation to treatment or control groups was done randomly.
Blinding	Blinding was used in microscope imaging, CFU determination on agar plates. Blinding is not relevant to the rest experiments since no elements that might be influenced by bias from the investigator or observer. Matching samples were collected and analyzed under the same conditions.

Reporting for specific materials, systems and methods

We require information from authors about some types of materials, experimental systems and methods used in many studies. Here, indicate whether each material, system or method listed is relevant to your study. If you are not sure if a list item applies to your research, read the appropriate section before selecting a response.

Materials & experimental systems

n/a	Involved in the study
☐	☒ Antibodies
☐	☒ Eukaryotic cell lines
☒	☐ Palaeontology and archaeology
☐	☒ Animals and other organisms
☒	☐ Clinical data
☒	☐ Dual use research of concern

Methods

n/a	Involved in the study
☒	☐ ChIP-seq
☐	☒ Flow cytometry
☒	☐ MRI-based neuroimaging

Antibodies

Antibodies used

Horseradish peroxidase-linked anti-mouse IgG antibody (dilution 1:5000; #7076V), Horseradish peroxidase -linked anti-rabbit IgG antibody (dilution 1:5000; #7074V), vimentin rabbit monoclonal D21H3 antibody (dilution 1:1000; #5741), MEK1/2 mouse monoclonal L38C12 antibody (dilution 1:1000; #4694), phospho-MEK1/2 (Ser217/221) rabbit monoclonal 41G9 antibody (dilution 1:1000; #9154), p44/42 MAPK (Erk1/2) (137F5) Rabbit mAb (dilution 1:1000; #4695), Phospho-p44/42 MAPK (Erk1/2) (Thr202/Tyr204) (D13.14.4E) XP® Rabbit mAb (dilution 1:1000; #4370), LAMP1 rabbit monoclonal C54H11 antibody (dilution 1:1000; #3243) and LAMP1 rabbit monoclonal D2D11 antibody (dilution 1:100; #9091) were purchased from Cell Signaling Technology (US). Alexa Fluor 488 goat anti-rabbit (dilution 1:1000; #A11008), Alexa Fluor 568 goat anti-rabbit (dilution 1:1000; #A11011), Alexa Fluor 488 goat anti-mouse (dilution 1:1000; #A11001), Alexa Fluor 555 goat anti-mouse (dilution 1:1000; #A21422), Alexa Fluor 568 goat anti-chicken (dilution 1:1000; #A11041), Alexa Fluor 633 goat anti-chicken (dilution 1:1000; #A21103), Alexa Fluor 647 goat anti-mouse (dilution 1:1000; #A21235) and Alexa Fluor 488 goat anti-rabbit (dilution 1:1000; #A21244) were purchased from Invitrogen (US). Monoclonal Anti-Green Fluorescent Protein (GFP) antibody produced in mouse (dilution 1:1000; #G1546), Polyclonal GroEL antibody (dilution 1:80,000; #G6532), GAPDH rabbit monoclonal antibody (dilution 1:5000; #G8795) and Monoclonal Anti-FLAG(M2) antibody (dilution 1:1000; #F1804) were purchased from Sigma-Aldrich (US). Vimentin chicken polyclonal antibody (dilution 1:1000; #ab24525) were purchased from Abcam (UK). Polyclonal CDC42 Rabbit (dilution 1:1000; #10155-1-AP) were purchased from Fisher Scientific (UK).

Validation

Horseradish peroxidase-linked anti-mouse IgG antibody (#7076V)
https://www.cellsignal.cn/products/secondary-antibodies/anti-mouse-igg-hrp-linked-antibody/7076
Horseradish peroxidase -linked anti-rabbit IgG antibody (#7074V)
https://www.cellsignal.cn/products/secondary-antibodies/anti-rabbit-igg-hrp-linked-antibody/7074
vimentin rabbit monoclonal D21H3 antibody (#5741)
https://www.cellsignal.cn/products/primary-antibodies/vimentin-d21h3-xp-rabbit-mab/5741
MEK1/2 mouse monoclonal L38C12 antibody (#4694)
https://www.cellsignal.cn/products/primary-antibodies/mek1-2-l38c12-mouse-mab/4694
phospho-MEK1/2 (Ser217/221) rabbit monoclonal 41G9 antibody (#9154)
https://www.cellsignal.cn/products/primary-antibodies/phospho-mek1-2-ser217-221-41g9-rabbit-mab/9154
p44/42 MAPK (Erk1/2) (137F5) Rabbit mAb (#4695)
https://www.cellsignal.cn/products/primary-antibodies/p44-42-mapk-erk1-2-137f5-rabbit-mab/4695
Phospho-p44/42 MAPK (Erk1/2) (Thr202/Tyr204) (D13.14.4E) XP® Rabbit mAb (#4370)
https://www.cellsignal.cn/products/primary-antibodies/phospho-p44-42-mapk-erk1-2-thr202-tyr204-d13-14-4e-xp-rabbit-mab/4370
LAMP1 rabbit monoclonal C54H11 antibody (#3243)
https://www.cellsignal.cn/products/primary-antibodies/lamp1-c54h11-rabbit-mab/3243
LAMP1 rabbit monoclonal D2D11 antibody (#9091)
https://www.cellsignal.cn/products/primary-antibodies/lamp1-d2d11-xp-rabbit-mab/9091
Alexa Fluor 488 goat anti-rabbit (#A11008)
https://www.thermofisher.cn/order/genome-database/dataSheetPdf?
producttype=antibody&productsubtype=antibody_secondary&productId=A-11008&version=256
Alexa Fluor 568 goat anti-rabbit (#A11011)
https://www.thermofisher.cn/order/genome-database/dataSheetPdf?
producttype=antibody&productsubtype=antibody_secondary&productId=A-11011&version=256
Alexa Fluor 488 goat anti-mouse (#A11001)
https://www.thermofisher.cn/order/genome-database/dataSheetPdf?
producttype=antibody&productsubtype=antibody_secondary&productId=A-11001&version=256
Alexa Fluor 555 goat anti-mouse (#A21422)
https://www.thermofisher.cn/order/genome-database/dataSheetPdf?
producttype=antibody&productsubtype=antibody_secondary&productId=A-21422&version=256
Alexa Fluor 568 goat anti-chicken (#A11041)
https://www.thermofisher.cn/order/genome-database/dataSheetPdf?
producttype=antibody&productsubtype=antibody_secondary&productId=A-11041&version=256
Alexa Fluor 633 goat anti-chicken (#A21103)
https://www.thermofisher.cn/order/genome-database/dataSheetPdf?
producttype=antibody&productsubtype=antibody_secondary&productId=A-21103&version=256
Alexa Fluor 647 goat anti-mouse (#A21235)
https://www.thermofisher.cn/order/genome-database/dataSheetPdf?
producttype=antibody&productsubtype=antibody_secondary&productId=A-21235&version=256
Alexa Fluor 488 goat anti-rabbit (#A21244)
https://www.thermofisher.cn/order/genome-database/dataSheetPdf?
producttype=antibody&productsubtype=antibody_secondary&productId=A-21244&version=256
Monoclonal Anti-Green Fluorescent Protein (GFP) antibody produced in mouse (#G1546)

https://www.sigmaaldrich.cn/deepweb/assets/sigmaaldrich/product/documents/426/225/g1546dat-mk.pdf
Polyclonal GroEL antibody (#G6532)
https://www.sigmaaldrich.cn/deepweb/assets/sigmaaldrich/product/documents/767/367/g6532dat.pdf
GAPDH rabbit monoclonal antibody (#G8795)
https://www.sigmaaldrich.cn/deepweb/assets/sigmaaldrich/product/documents/375/053/g8795dat.pdf
Monoclonal Anti-FLAG(M2) antibody (#F1804)
https://www.sigmaaldrich.cn/specificationsheets/469/360/F18045MG_SIGMA_.pdf
Vimentin chicken polyclonal antibody (#ab24525)
https://www.abcam.cn/vimentin-antibody-ab24525.html
Polyclonal CDC42 Rabbit (#10155-1-AP)
https://www.fishersci.co.uk/shop/products/cdc42-rabbit-anti-human-mouse-rat-polyclonal-proteintech2/16826353

Eukaryotic cell lines

Policy information about cell lines and Sex and Gender in Research

Cell line source(s)	Human osteosarcoma U2OS cells were gifts from Pekka Lappalainen (University of Helsinki), mouse macrophage cell line RAW 264.7 and mouse embryonic fibroblasts cell line MEFs were gifts from John Eriksson (University of Turku), and mouse fibroblast L929 cells were gifts from Li Yu (Tsinghua University), human HEK293T cells were gifts from Jin Zhong (Institut Pasteur of Shanghai, Chinese Academy of Sciences).
Authentication	Each cell line used were authenticated by the host lab by comparing the phenotypes including growth, upon stimuli, ect.
Mycoplasma contamination	We detected mycoplasma contamination using Mycoplasma Stain Assay Kit (#C0296, Beyotime, CN) to make sure negative contamination.
Commonly misidentified lines (See ICLAC register)	No commonly misidentified cell lines were used in the study.

Animals and other research organisms

Policy information about studies involving animals; ARRIVE guidelines recommended for reporting animal research, and Sex and Gender in Research

Laboratory animals	8 weeks old C57/B6j mice were used in this study.
Wild animals	The study did not involve wild animals.
Reporting on sex	Male mice were used in this study, and 5 mice per group were used.
Field-collected samples	The study did not involve samples collected from the field.
Ethics oversight	All animals were used following institutional ethics requirements under the animal user permit (No. P2021014) approved by the Institute of Pasteur Animal Care Committee.

Note that full information on the approval of the study protocol must also be provided in the manuscript.

Flow Cytometry

Plots

Confirm that:

☒ The axis labels state the marker and fluorochrome used (e.g. CD4-FITC).

☒ The axis scales are clearly visible. Include numbers along axes only for bottom left plot of group (a 'group' is an analysis of identical markers).

☒ All plots are contour plots with outliers or pseudocolor plots.

☒ A numerical value for number of cells or percentage (with statistics) is provided.

Methodology

Sample preparation	Cells were digested by trypsin with multiple time points. Cells were then fixed with 4% PFA and resuspended in PBS.
Instrument	Flow cytometry (Celesta, BD, US)
Software	Flowjo10.5
Cell population abundance	5x10^4 cells/sample were recorded by the instrument.

Gating strategy

mCherry tagged bacteria infected cell were defined as PE channel positive cells.

☒ Tick this box to confirm that a figure exemplifying the gating strategy is provided in the Supplementary Information.
